# Vaccination willingness, vaccine hesitancy, and estimated coverage of SARS‐CoV‐2 vaccine among healthcare workers in Tanzania: A call for action

**DOI:** 10.1002/iid3.1126

**Published:** 2023-12-26

**Authors:** Suzan Joseph Kessy, Tingting Wei, Yiguo Zhou, Wan‐Xue Zhang, Fadhlun M. Alwy Al‐Beity, Shan‐Shan Zhang, Juan Du, Fuqiang Cui, Qing‐Bin Lu

**Affiliations:** ^1^ Department of Laboratory Science and Technology & Vaccine Research Center School of Public Health, Peking University Beijing China; ^2^ Training Division Infection Control African Network (ICAN) Cape Town South Africa; ^3^ Department of Health Policy and Management, School of Public Health Peking University Beijing China; ^4^ Department of Epidemiology and Biostatistics, School of Public Health Peking University Beijing China; ^5^ Department of Obstetrics and Gynaecology, School of Medicine Muhimbili University of Allied Sciences (MUHAS) Dar es Salaam Tanzania; ^6^ Global Center for Infectious Disease and Policy Research & Global Health and Infectious Diseases Group Peking University Beijing China; ^7^ Key Laboratory of Epidemiology of Major Diseases (Peking University), Ministry of Education Beijing China

**Keywords:** coverage, COVID‐19, healthcare workers, hesitancy, SARS‐CoV‐2 vaccine, willingness

## Abstract

**Background:**

The global COVID‐19 pandemic presented an immense obstacle to public health, with vaccination emerging as a crucial measure to curb transmission. This study aimed to evaluate the willingness, hesitancy, and coverage of SARS‐CoV‐2 vaccines among healthcare workers (HCWs) in Tanzania and reveal their concerns about SARS‐CoV‐2 vaccines and the reasons that might prevent them from getting vaccinated.

**Methods:**

We conducted a cross‐sectional study using an anonymous online survey from October to November 2022. The multivariate logistic regression model explored the factors associated with SARS‐CoV‐2 vaccine willingness, hesitancy, and coverage.

**Results:**

The study included 560 HCWs, with the largest group being doctors (47.9%), followed by nurses (26.9%) and other HCWs (25.2%). A total of 70.5% of HCWs reported being vaccinated against SARS‐CoV‐2. The primary driver for SARS‐CoV‐2 vaccination was collective responsibility. A total of 81.4% of HCWs reported being willing to accept SARS‐CoV‐2 vaccines, while 62.5% of HCWs reported vaccine hesitancy. HCWs with higher educational qualifications were likelier to take the vaccine, while the respondents aged 18–30 years had the highest SARS‐CoV‐2 vaccination refusal (71.9%). We also investigated the role of HCWs as a source of information to promote COVID‐19 vaccine uptake. 79.4% of HCWs provided information and advice on SARS‐CoV‐2 vaccines.

**Conclusion:**

To increase vaccine acceptance among HCWs and the general population, targeted messaging is needed to deliver transparent information on vaccine safety, efficacy, and development.

## Graphical abstract

Our research paper aims to shed light on SARS‐Cov‐2 vaccination coverage, willingness, and hesitancy. Additionally, we point out the underlying factors contributing to vaccine hesitancy and willingness among HCWs in Tanzania.

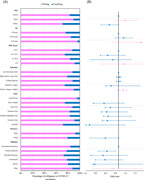

## 1 INTRODUCTION

Vaccination significantly reduces infectious diseases and is recognized for its role in disease control, elimination, and eradication.^1^ Numerous viral vaccines, including measles, influenza, rubella, and hepatitis, have saved over 50 million lives from 2000 to 2019.^2^ However, the success of vaccination depends on achieving sufficient vaccination coverage.^3^ In particular, the SARS‐CoV‐2 vaccine is of utmost importance in mitigating the COVID‐19 pandemic, which has caused over six million deaths and infected hundreds of millions worldwide as of May 2023, resulting in significant morbidity.^4^

The promotion and advocacy of vaccination primarily targeted healthcare workers (HCWs). The World Health Organization (WHO) prioritized the vaccination of HCWs, as they play a crucial role in managing the pandemic and face a high risk of exposure to SARS‐CoV‐2.^3^ Ensuring the safety of HCWs is crucial to maintaining disease prevention and control and preventing virus transmission during frequent social interactions. Moreover, given that SARS‐CoV‐2 vaccination is largely voluntary,^5^ HCWs are perceived as the most trusted source of information regarding therapeutics and vaccines, aiding vaccine decision‐making.^6^ However, one research has revealed relatively high levels of vaccine hesitancy among HCWs worldwide.^7^ HCWs’ hesitancy continues to undermine vaccine confidence, contribute to the community losing confidence in SARS‐CoV‐2 vaccination, and increase vaccine hesitancy among the general population.^1^, ^7^ Therefore, understanding and addressing the drivers of SARS‐CoV‐2 vaccine hesitancy among HCWs is pivotal to improving vaccine uptake and curbing the burden of COVID‐19. In addition, the knowledge can further help in other vaccine‐preventable diseases.^8^

The reasons for vaccine hesitancy among HCWs are multifaceted and depend on various factors, such as context, time, location, and vaccine type of vaccine.^9^ Therefore, it is crucial to consider obstacles to vaccination unique to specific settings and subgroups of the HCWs.^7^

Industrialized nations tend to be ahead of low‐income countries like Tanzania when adopting new vaccines, which are often designed to meet the demands of the commercial market.^10^ However, efforts have been made to ensure equitable SARS‐CoV‐2 vaccines through initiatives such as the COVID‐19 Vaccines Global Access (COVAX) program. Tanzania received its first SARS‐CoV‐2 vaccine in July 2021, and more than 40 million doses have been received through COVAX and other partners as of March 2023.^11^, ^12^, ^13^ Despite these efforts, the Tanzanian government still faced significant challenges in managing the pandemic, ranging from obtaining vaccines to overcoming vaccine hesitancy among the population and increasing vaccine demand and uptake.^14^ Tanzania Medicines and Medical Devices Authority (TMDA) has authorized the marketing of vaccines, which had received a prior recommendation from the Ministry of Health. These included Janssen‐Ad26.COV2.S, Pfizer/BioNTech‐BNT162b2/COMIRNATY Tozinameran, Moderna‐mRNA‐1273, Sinopharm‐SARS‐CoV‐2 Vaccine (Vero cell), inactivated (InCoV), and Sinovac‐COVID‐19 Vaccine (Vero Cell), Inactivated/CoronavacTM.^15^ As of March 2023, More than 38M vaccine doses have been administered, representing 58% of the population and 55% being fully vaccinated.^16^ However, more than the availability of vaccines alone is needed to guarantee sufficient population vaccination, as vaccine hesitancy remains a challenge.^17^

Vaccine hesitancy among HCWs did not start with COVID‐19. Several pre‐COVID‐19 works examined HCWs' vaccine hesitancy against vaccine‐preventable diseases,^18^, ^19^, ^20^, ^21^ but mainly in developed countries, and only a few of them are focused on Africa.^22^ Given the impact of COVID‐19 on the public and the rise of COVID‐19 HCWs infection in Africa,^23^ discussions on HCW vaccine attitudes have increased in Africa,^8^, ^24^, ^25^, ^26^, ^27^, ^28^, ^29^, ^30^, ^31^, ^32^ with only one article analysing vaccine hesitancy in Tanzania.^8^ Therefore, this study aimed to shed light on overall SARS‐CoV‐2 vaccine hesitancy, willingness, and coverage among HCWs in Tanzania and explore the nature of their concerns, perceptions of vaccine‐related information, and perceived role in responding to vaccine hesitancy. Ultimately, this study informed efforts to promote and improve vaccine coverage in the general population.

## 2 METHODS

### 2.1 Study design and population

A cross‐sectional online survey was conducted among HCWs in Tanzania using a structured questionnaire from October to November 2022. Individuals aged 18 years or older were eligible to participate in the study. The study only included doctors, nurses, medical technicians, hospital administrators, pharmacists, and medical students. The term “doctors” encompasses various types of physicians, including physician assistants and resident physicians. Similarly, the category “nurses” includes nurse practitioners and other advanced practice nursing roles. Hospital administrators are individuals responsible for the administration of a hospital, such as executive directors or managerial personnel, rather than nonclinical support staff like secretaries or administrative assistants. Only students enrolled in Doctor of Medicine (MD) programs were included in the study. Other healthcare professions students, such as nursing students, were excluded. Former or emeritus HCWs were allowed to participate. Participants had to have both resided and worked in Tanzania during the survey. Additionally, subjects with questionnaire responses containing conflicting answers or missing information were excluded from the analysis. Participants were recruited online. The selection procedure was based on convenience sampling among HCWs. HCWs were conveniently sampled in a snowball sampling procedure. The sample size calculations were determined with a rate of 30% as the estimated proportion of subjects with vaccine hesitancy.^33^ To achieve the research objectives with a 95% confidence interval and 5% absolute precision on both sides, 399 HCWs were required. The questionnaire was shared on social media platforms like WhatsApp, Twitter, Instagram, and LinkedIn and biweekly reminders were sent. On average, participants could complete the questionnaire using a mobile phone or computer, which took around 5 min. Wen Juan Xing (https://www.wjx.cn), China's largest online survey platform, was used to distribute questionnaires. The response rate to the survey could not be estimated because it was distributed electronically to all HCWs who could access social networks.

### 2.2 Data collection

The structured questionnaire (Table S1) contained seven sections: the first section focused on demographic information (age, sex, profession, education, residence, and insurance). The second section was on SARS‐CoV‐2 infection and preventive measures. The third section was on SARS‐CoV‐2 vaccination coverage, willingness and hesitancy, and the underlying reasons for their stance. The fourth section inquired about HCWs' counseling behavior and confidence in recommending SARS‐CoV‐2 vaccines to the public. The fifth section focused on vaccine preference and the reasons for their selection. The sixth section was on the vaccination mandate, and the last section collected data on SARS‐CoV‐2 vaccine information.

### 2.3 Vaccination coverage

Vaccine coverage measured the number of HCWs who had received at least one dose of the SARS‐CoV‐2 vaccine. Those who answered “Yes” to the question, “Have you received the SARS‐CoV‐2 vaccine?” were recorded as being vaccinated. HCWs are considered fully vaccinated for SARS‐CoV‐2 if they have received the second dose in a two‐dose series (Pfizer‐BioNTech or Moderna or other vaccine authorized by the WHO), or they have received a single‐dose vaccine (Johnson and Johnson [J&J]/Janssen).^34^

### 2.4 Vaccination willingness

Participants were considered “willing” if they had either received at least one dose of the SARS‐CoV‐2 vaccine or expressed their intention to do so. The willingness to receive the vaccine was evaluated through the question “Have you received the SARS‐CoV‐2 vaccine?” and was scored as a “yes” response. Those who stated, “I would get vaccinated as soon as possible” or “were willing to get vaccinated, but wait” to the question, “If a SARS‐CoV‐2 vaccine was available to you, would you get it?” were also considered “willing.”

### 2.5 Vaccine hesitancy

Vaccine hesitancy was characterized as reluctance, postponement, or outright refusal to receive the SARS‐CoV‐2 vaccine for reasons unrelated to vaccine availability or contraindication. It was assessed by answering “no” to the question “Have you received the SARS‐CoV‐2 vaccine?” and “yes” to the question “Were you initially hesitant?” followed by responding with “willing to get vaccinated, but wait”, “would not get vaccinated even if the vaccine was available”, or “not sure if I would get vaccinated” to the query “If SARS‐CoV‐2 vaccine were available to you, would you get it?”.

### 2.6 Statistical analysis

Descriptive and frequency statistics were calculated for individual questionnaire items. The body mass index (BMI) was calculated based on their self‐reported height and weight, and standard WHO criteria of adult BMI grouping was applied as follows: underweight (BMI <18.5 kg/m^2^), normal weight (BMI: 18.5–24.9 kg/m^2^), overweight (BMI: 25.0–29.9 kg/m^2^), and obese (BMI ≥30 kg/m^2^). The variable of age was stratified into four groups according to distribution: 18–29 years, 30–39 years, 40–49 years, and 50 years and above. HCWs were classified into three categories: doctors, nurses, and other HCWs, and the regions were re‐classified into six zones based on the Bank of Tanzania categorization,^35^ including northern, central, lake, southern, southern highlands, and Zanzibar, as well as the Dar es Salaam zone (Table S2). The Chi‐square test was used to compare the vaccine coverage, willingness and hesitancy to HCWs’ demographic and background characteristics and attitudes. The multivariable logistic regression model evaluated how demographic and attitudinal factors influence respondents' willingness or hesitancy to receive the SARS‐CoV‐2 vaccine. All statistical analyses were performed by R version 4.1.2 (R Development Core Team) and Excel 2019. A two‐sided *p* < .05 was considered statistically significant.

### 2.7 Ethical considerations

The National Health Research Ethics Review Committee–Tanzania approved the research plan under Certificate No. NIMR/HQ/R.8a/Vol.IX/4122. A summary of the research was included at the beginning of the questionnaire. To ensure consent, the first question in the questionnaire asked participants if they were sufficiently informed about the study and if they wanted to participate voluntarily. Only those who clicked “Yes” could continue answering the questions.

## 3 RESULTS

### 3.1 Background characteristics

In total, 560 HCWs completed the questionnaire. Table 1 and Table S3 present the background characteristics and historical context of SARS‐CoV‐2 infection by three categories of professions and by all six professions, respectively. The distribution of participants across all six zones was notably unequal, with most respondents originating from Dar es Salaam zone (43%, 241/560), followed by lake (21%, 120/560), northern (13%, 75/560), central (8%, 42/560), southern highlands (7%, 41/560), and Zanzibar zone (1%, 8/560). Among the participants, 47.9% (268/560) were medical doctors, 26.9% (151/560) were nurses, and 25.2% (141/560) were other HCWs. Most respondents (57.5%, 322/560) were males with a mean (*SD*) age of 33 (±8) years. Most participants (85.9%, 481/560) perceived their health as good or excellent. However, a small proportion (14.1%, 79/560) reported underlying conditions. Over half of the HCWs were overweight or obese (55.8%, 313/560). With regards to SARS‐CoV‐2 infection, 31.4% (176/560) reported that they had contracted COVID‐19, 57.7% (323/560) had not, and 10.9% (61/560) did not know whether they had acquired COVID‐19, of the individuals who disclosed having contracted COVID‐19, 55.6% (98/176) reported receiving medical care without being admitted to the hospital.

**Table 1:** Characteristics of healthcare workers by profession.

Characteristics, *n* (%)	Total (*N* = 560)	Healthcare workers/profession		
		Doctors (*n* = 268)	Nurse (*n* = 151)	Other HCWs (*n* = 141)
**Sex**				
Female	238 (42.5)	166 (61.9)	74 (49.0)	82 (58.2)
Male	322 (57.5)	102 (38.1)	77 (51.0)	59 (41.8)
**Age group**				
Under 30 years	242 (43.2)	63 (23.5)	94 (62.3)	85 (60.3)
30–49 years	294 (52.9)	190 (70.9)	53 (35.1)	53 (37.6)
Above 50 years	24 (3.9)	15 (5.6)	4 (2.6)	3 (2.1)
**BMI**				
≤18.5	23 (4.1)	9 (3.4)	7 (4.6)	7 (5.0)
18.5–24.9	224 (40.0)	93 (34.7)	67 (44.4)	64 (45.4)
25–29.9	198 (35.4)	111 (41.4)	48 (31.8)	39 (27.7)
>30	115 (20.5)	55 (20.5)	29 (19.2)	31 (22.0)
**Insurance**				
Yes	427 (76.3)	224 (83.6)	107 (70.9)	96 (68.1)
No	125 (22.3)	41 (15.3)	42 (27.8)	42 (29.8)
Not sure	8 (1.4)	3 (1.1)	2 (1.3)	3 (25.0)
**Education**				
Never	1 (0.2)	0 (0)	0 (0)	1 (0.7)
Less than high school	8 (1.4)	0 (0)	6 (4.0)	2 (1.4)
High school or equivalent (e.g., college certificate)	33 (5.9)	1 (0.4)	14 (9.3)	18 (12.8)
College diploma	122 (21.8)	13 (4.9)	79 (52.3)	30 (21.3)
Bachelor's degree	260 (46.4)	148 (55.2)	42 (27.8)	70 (49.6)
Master's degree or higher	136 (24.3)	106 (39.6)	10 (6.6)	20 (14.2)
**Residency**				
Urban	458 (81.8)	230 (85.8)	116 (76.8)	112 (79.4)
Rural	102 (18.2)	38 (14.2)	35 (23.6)	29 (20.6)
**Residency (zone)**				
Northern	75 (13.4)	32 (42.7)	27 (36.0)	16 (21.3)
Central	42 (7.5)	24 (57.1)	(31.0)	5 (11.9)
Lake	120 (21.4)	46 (38.3)	51 (42.5)	23 (19.2)
Southern	33 (5.9)	14 (42.4)	16 (48.5)	3 (9.1)
Southern highlands	41 (7.3)	15 (36.6)	11 (26.8)	15 (36.6)
Zanzibar	8 (1.4)	5 (62.5)	0 (0)	3 (37.5)
Dar es Salaam	241 (43.0)	132 (54.5)	33 (13.7)	76 (31.5)
**Healthcare setting**				
Government institution	309 (55.2)	170 (63.4)	78 (51.7)	61 (43.3)
Private institution	167 (29.8)	71 (26.5)	50 (33.1)	46 (32.6)
Individual business	11 (2.0)	6 (2.2)	2 (1.3)	3 (2.1)
Unemployed	52 (9.3)	9 (3.4)	17 (11.3)	26 (18.4)
Retiree	5 (0.9)	3 (1.1)	0 (0.0)	2 (1.4)
Other	16 (2.9)	9 (3.4)	4 (2.6)	0 (0)
**Healthcare setting**				
Community health center	76 (13.6)	33 (12.3)	23 (15.2)	20 (14.3)
Dispensary	31 (5.5)	10 (3.7)	12 (7.9)	9 (6.4)
District hospital	77 (13.8)	28 (10.4)	38 (25.2)	11 (7.9)
Regional hospital	59 (10.6)	35 (13.1)	20 (13.2)	4 (2.9)
Referral and specialized hospital	96 (17.2)	63 (23.5)	15 (9.9)	18 (12.9)
National hospital	51 (9.1)	31 (11.6)	10 (6.6)	10 (7.1)
Other	118 (21.1)	59 (22.0)	16 (10.6)	43 (30.7)
Unemployed	52 (9.3)	9 (3.4)	17 (11.3)	26 (18.4)
**Chronic disease**				
Yes	79 (13.6)	38 (14.2)	18 (11.9)	20 (14.2)
No	481 (86.4)	230 (85.8)	133 (88.1)	121 (86.4)
**History of COVID‐19 infection**				
Yes	176 (31.4)	105 (39.2)	30 (19.9)	41 (29.1)
No	323 (57.7)	128 (47.8)	111 (73.5)	84 (59.6)
I don't know	61 (10.9)	35 (13.1)	10 (6.6)	16 (11.3)

Abbreviations: BMI, body mass index; HCW, healthcare worker.

### 3.2 Vaccination coverage

**Figure 1:**
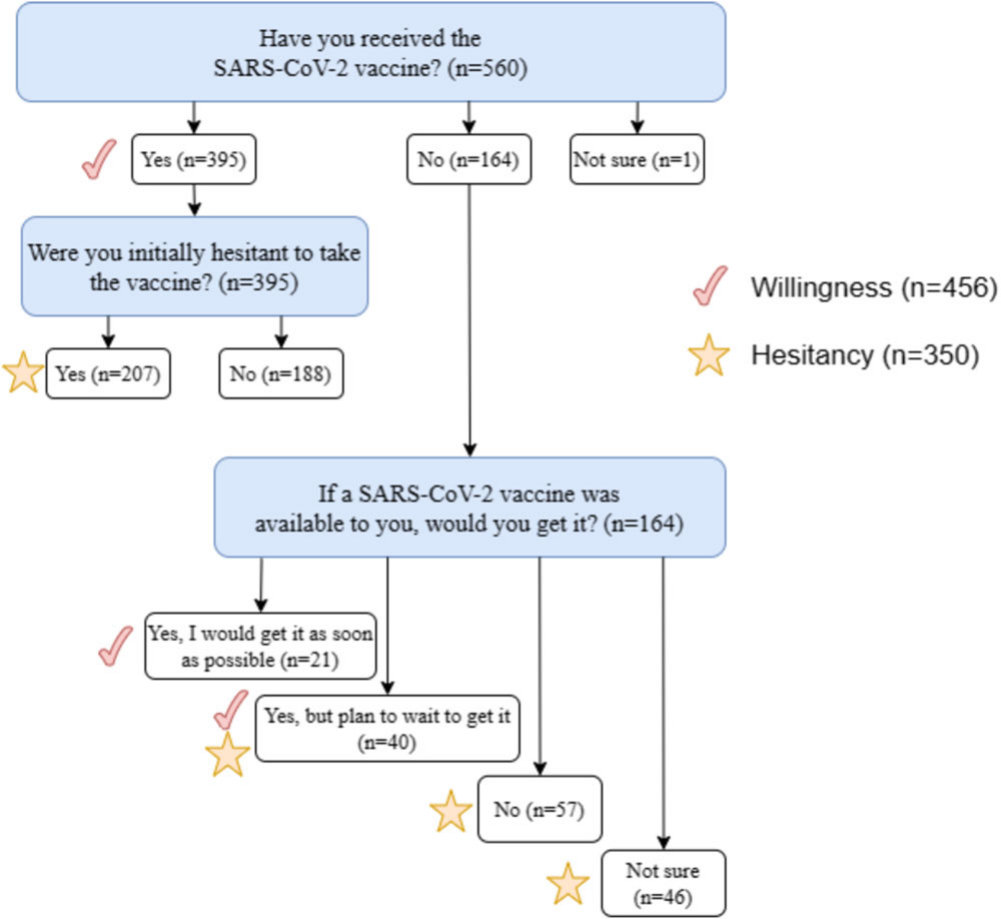
SARS‐CoV‐2 vaccination hesitancy and willingness grouping flow chart.

Overall, 70.5% (395/560) of the participants indicated that they had received at least one dose of the SARS‐CoV‐2 vaccine at the time of this survey with 69.1% (387/560) being fully vaccinated (Figure 1 and Table S4). A total of 29.2% (164/560) had not received any dose of the SARS‐CoV‐2 vaccine, and one (0.17%) participant was unsure whether they were vaccinated. Janssen‐Ad26.COV2.S, a one‐dose vaccine, was the most widely used vaccine (82.0%, 324/395) (Figure S1).

**Figure 2:**
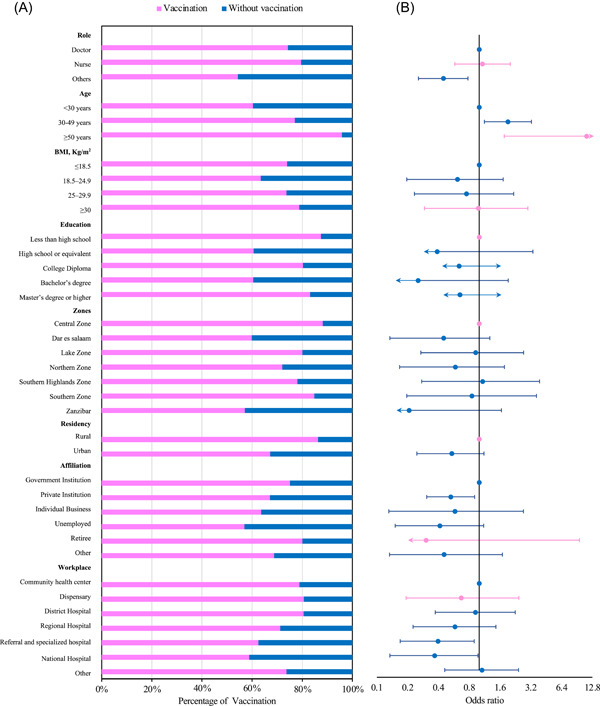
SARS‐CoV‐2 vaccination coverage among HCWs in Tanzania. (A) Rate of SARS‐CoV‐2 vaccination among HCWs. (B) Odds ratios comparing vaccination among HCWs with different characteristics. HCW, healthcare worker.

Certain demographic variables exhibited distinct associations with the likelihood of SARS‐CoV‐2 vaccination. Specifically, older individuals displayed a notably higher rate of vaccination uptake (95.8%, 23/24) (*p* < .001). Similarly, individuals with BMI ≥30 kg/m^2^ showed a propensity towards vaccination (78.3%, 90/114) (*p* = .015). Likewise, a substantial proportion of those with an education level below high school were vaccinated (87.5%, 7/8) (*p* < .001). Furthermore, participants residing in the central zone exhibited a considerable vaccination rate of 88.1% (37/42) (*p* < .001), while those dwelling in rural areas displayed a prominent rate of vaccination (86.3%, 88/102) (*p* < .001). Health center employees manifested a significant inclination toward vaccination, with 78.9% (60/67) having received it, and those occupying the role of nurses also displayed a pronounced vaccination rate of 79.5% (120/151) (*p* < .001) as shown in Figure 2A and Table S5. All potential variables were incorporated into the logistic regression model. The multivariate analysis confirmed that SARS‐CoV‐2 vaccination could be associated with the profession, age, affiliation and workplace, with those not being doctors or nurses, those aged below 30 years, those working in private institutions and those working in referral and specialized hospitals and national hospitals being the least vaccinated (Figure 2B and Table S6).

To gain a deeper understanding of HCWs’ willingness to get vaccinated, those vaccinated were asked about what influenced their decision. For example, 17.2% (68/395) indicated that they did more research on SARS‐CoV‐2 vaccines, 37.9% (150/395) believed that they had a responsibility as HCWs to lead by example, and 7.1% (28/395) were motivated by fear of increasing SARS‐CoV‐2 infections and deaths. We grouped participants’ responses into three categories based on the SAGE Vaccine Hesitancy Matrix: vaccine‐related attitude, contextual influence, and individual or group influence. Table 2 provides a detailed breakdown of the reasons that influenced HCWs to be vaccinated.

**Table 2:** Factors that influenced HCWs to receive vaccination.

Reasons, *n* (%)	Total (*N* = 395)	Healthcare workers/profession		
		Doctors (*n* = 199)	Nurses (*n* = 120)	Other HCWs (*n* = 76)
**Vaccine related attitude**				
Offered the vaccine that I was more comfortable	77 (19.5)	33 (16.6)	27 (22.5)	17 (22.4)
**Contextual influence**				
I was satisfied with the information I saw in the media (radio, TV, social media)	8 (2.0)	5 (2.5)	1 (0.8)	2 (2.6)
My spiritual/religious leader advised me)	1 (0.3)	1 (0.5)	0 (0)	0 (0)
Persuaded by government/public health authorities)	19 (4.8)	5 (2.5)	6 (5.0)	8 (10.5)
Noticed that a personality (social influencer/public or otherwise) took it)	1 (0.3)	1 (0.5)	0 (0)	0 (0)
I was forced by my employer/family members/didn't have a choice)	8 (2.0)	4 (2.0)	2 (1.7)	2 (2.6)
**Individual or group influence**				
Felt more comfortable because several people took it	4 (1.0)	3 (1.5)	0 (0)	1 (1.3)
I did more research on COVID‐19 vaccines	67 (17.0)	45 (22.6)	12 (10.0)	10 (13.2)
I am a healthcare worker I had to lead by example	150 (38.0)	66 (33.2)	60 (50.0)	24 (31.6)
Frightened by the increased infections/deaths	28 (7.1)	17 (8.5)	6 (5.0)	5 (6.6)
My circumstances changed (such as being pregnant or other medical or personal factors)	3 (0.8)	2 (1.0)	1 (0.8)	0 (0)
I was influenced by my colleagues)	4 (1.0)	1 (0.5)	1 (0.8)	2 (2.6)
Consulted family and friends)	9 (2.3)	3 (1.5)	2 (1.7)	4 (5.3)
Other	16 (4.1)	13 (6.5)	2 (1.7)	1 (1.3)

Abbreviation: HCW, healthcare worker.

### 3.3 Vaccine willingness

For vaccination willingness, those HCWs who reported being vaccinated (70.5%, 395/560), unvaccinated but reported they would get vaccinated as soon as possible 12.8% (21/164), and unvaccinated and willing to get vaccinated but plan to wait to get it 24.3% (40/164) were included (Figure 1). Therefore, 81.4% (456/560) of participants reported willingness to accept the COVID‐19 vaccination, and 18.6% (106/560) were reportedly unwilling to receive the COVID‐19 vaccination.

**Figure 3:**
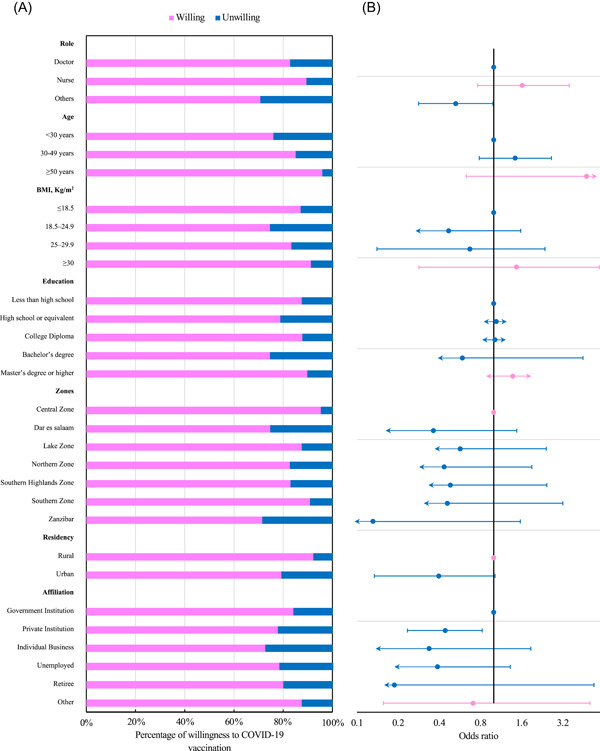
Distribution of willingness to accept the SARS‐CoV‐2 vaccine among HCWs in Tanzania. (A) Rate of willingness to accept SARS‐CoV‐2 vaccination among HCWs. (B) Odds ratios comparing the rate of SARS‐CoV‐2 vaccine willingness among HCWs with different characteristics. HCW, healthcare worker.

The willingness of HCWs to receive the SARS‐CoV‐2 vaccine based on their background and demographic characteristics are shown in Figure 3A and Table S7. The rate of willingness to accept the SARS‐CoV‐2 vaccine was higher in participants above 50 years (95.8%, 23/24) (*p* = .006) in comparison to the other three age groups. HCWs with a Master's degree or higher were more willing to take SARS‐CoV‐2 vaccination than those with a diploma or lower level (89.7%, 122/136) (*p* < .001). Participants from rural areas (92.2%, 94/102) (*p* = .002) and those living in the central zone (95.2%, 40/42) (*p* = .003) reported higher SARS‐CoV‐2 vaccine willingness. Among HCWs, nurses (89.4%, 135/151) reported the highest rate of SARS‐CoV‐2 vaccine willingness, followed by doctors (82.8%, 222/268) and other HCWs (70.2%, 99/141). Those with a BMI above 30 kg/m^2^ had a high rate of SARS‐CoV‐2 vaccine willingness compared to the other four groups (90.4%, 104/115).

Logistic regression models were used to explore the SARS‐CoV‐2 vaccine willingness influencing factors and included all variables (Figure 3B and Table S8). The multivariate logistic regression model showed an unwillingness to accept the SARS‐CoV‐2 vaccination was associated with other HCWs (odds ratio [OR] = 0.528, 95% confidence interval [CI] = 0.282–0.986), affiliation with private institutions (OR = 0.442, 95% CI = 0.234–0.824), and workplace with dispensary (OR = 0.210, 95% CI = 0.049–0.874) and national hospital (OR = 0.258, 95% CI = 0.076–0.820). In comparison, no significant associations between the unwillingness to accept SARS‐CoV‐2 vaccination existed in sex, BMI, education, residency, and having pre‐existing conditions/disease in this study.

### 3.4 Vaccine hesitancy

**Figure 4:**
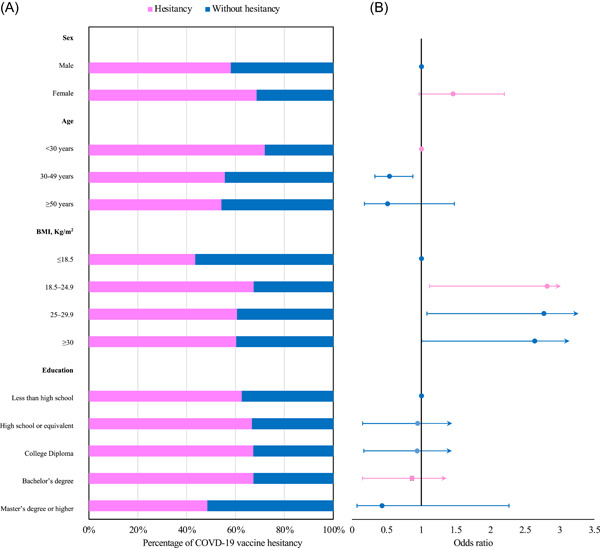
Distribution of t SARS‐CoV‐2 vaccine hesitancy among HCWs in Tanzania. (A) Rate of SARS‐CoV‐2 vaccine hesitancy among HCWs. (B) Odds ratios comparing the rate of SARS‐CoV‐2 vaccine hesitancy among HCWs with different characteristics. HCW, healthcare worker.

For vaccine hesitancy, those who were unvaccinated and planned to wait to get vaccinated (24.3%, 40/164), unvaccinated and would not get vaccinated even if the SARS‐CoV‐2 vaccine was available (34.7%, 57/164) and unvaccinated and were unsure if they would get vaccinated 28.0% (46/164) were included. Additionally, those HCWs who were vaccinated but were initially hesitant to SARS‐CoV‐2 vaccines (52.4%, 207/395) were included. Overall, 62.5% (350/560) reported SARS‐CoV‐2 vaccine hesitancy. Compared to the three groups of HCWs, other HCWs (68.1%, 96/141) exhibited the highest vaccine hesitancy. However, no significant association was found between vaccine hesitancy and the role of HCWs. Females had a higher vaccine hesitancy than males (68.5% vs. 58.1%, *p* = .015). Vaccine hesitancy decreased with an increase in age, with 49.7% (174/242) (*p* < .001) of HCWs in the age group below 30 years reporting higher SARS‐CoV‐2 vaccine hesitancy (Figure 4A and Table S9).

The multivariate logistic regression model included all variables (Figure 4B and Table S10). The model showed that participants aged 30–49 were less hesitant to SARS‐CoV‐2 vaccines (OR = 0.541, 95% CI = 0.330–0.879). Compared HCWs with BMI ≤18.5 kg/m^2^, those with a BMI of 18.5–24.9 kg/m^2^ (OR = 2.814, 95% CI = 1.119–7.29) and 25–29.9 (OR = 2.769, 95% CI = 1.080–7.312) and above 30 kg/m^2^ (OR = 2.637, 95% CI = 1.005–7.133) were more hesitant.

**Figure 5:**
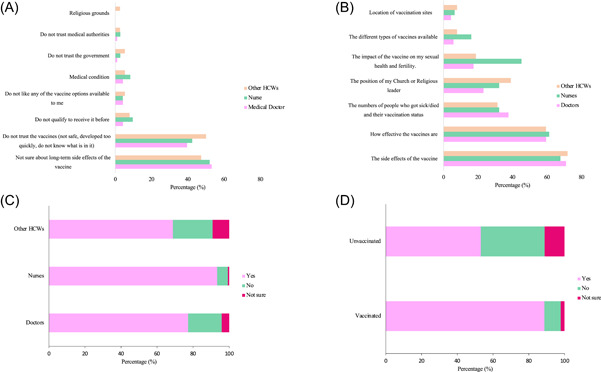
Reasons for vaccine hesitancy, information on SARS‐CoV‐2 vaccine and HCWs’ behavior in providing SARS‐CoV‐2 vaccine knowledge. (A) Reasons for vaccine hesitancy among vaccinated HCWs in Tanzania. (B) Information on SARS‐CoV‐2 vaccines needed to increase vaccine willingness among unvaccinated HCWs. (C) HCWs’ behavior in providing SARS‐CoV‐2 vaccine knowledge. (D) Comparison of vaccinated and unvaccinated HCWs’ behavior in providing SARS‐CoV‐2 vaccine knowledge. HCW, healthcare worker.

The most prevalent reason for SARS‐CoV‐2 vaccine hesitancy was concern about the short and long‐term effects of SARS‐CoV‐2 vaccines (68.9%, 113/164), followed by vaccine trust (12.2%, 20/164), information from the media (11.0%, 18/164) and safety (the risk of contracting COVID‐19 from the vaccine (10.4%, 17/164) of the SARS‐CoV‐2 vaccine (10.4%) (Table 3). These reasons were the same between hesitant vaccinated and unvaccinated HCWs. Figure 5A summarizes the reasons behind vaccine hesitancy among the vaccinated HCWs.

**Table 3:** Reasons that influenced unvaccinated HCWs’ hesitation.

Reasons, *n* (%)	Total (*N* = 164)	HCWs			*p* value
		Doctors (*n* = 69)	Nurses (*n* = 31)	Other HCWs (*n* = 64)	
**Vaccine related attitude**					
I am not sure about short or long‐term side effects of the vaccine	113 (68.9)	47 (68.1)	19 (61.3)	47 (73.4)	0.479
There is a risk of contracting COVID‐19 from the vaccine	17 (10.4)	7 (10.1)	2 (6.5)	8 (12.5)	0.710
People around me are vaccinated and their reaction after vaccination	7 (4.3)	2 (2.9)	1 (3.2)	4 (6.2)	0.693
Attitudes and techniques of vaccinators	3 (1.8)	2 (2.9)	1 (3.2)	0 (0)	0.410
I do not trust the vaccine (not safe, developed too quickly, do not know what is in it)	20 (12.2)	5 (7.2)	2 (6.5)	13 (20.3)	0.058
Previous experience or side effects of vaccination	13 (7.9)	6 (8.7)	2 (6.5)	5 (7.8)	1.000
I don't like any of the vaccine options available to me	9 (5.5)	3 (4.3)	4 (12.9)	2 (3.1)	0.162
I don't know where to go to get vaccinated	2 (1.2)	0 (0)	2 (6.5)	0 (0)	**0.035**
**Contextual influence**					
I will not take it on religious grounds	3 (1.8)	2 (2.9)	0 (0)	1 (1.6)	1.000
Beliefs, culture and traditions do not allow me to be vaccinated	1 (0.6)	0 (0)	0 (0)	1 (1.6)	0.579
Have heard (positive or negative) news from media (TV, Radio, and social media)	18 (11.0)	7 (10.1)	2 (6.5)	9 (14.1)	0.611
Recommendations from people around me	6 (3.7)	0 (0)	3 (9.7)	3 (4.7)	**0.028**
I do not trust the government/medical authorities here	20 (12.2)	5 (7.2)	2 (6.5)	13 (20.3)	0.058
**Individual and group influence**					
I believe it is a choice and I choose not to	60 (36.6)	23 (33.3)	15 (48.4)	22 (34.4)	0.315
No reason really, I just won't take it	16 (9.8)	6 (8.7)	5 (16.1)	5 (7.8)	0.429
It is not mandatory for my work	5 (3.0)	0 (0)	2 (6.5)	3 (4.7)	0.067
I am too busy and too much time is spent on the inoculation process	5 (3.0)	2 (2.9)	1 (3.2)	2 (3.1)	1.000
There are no rewards after vaccination	0 (0)	0 (0)	0 (0)	0 (0)	–
I'm not eligible to get a COVID‐19 vaccine. I have a medical reason that makes me ineligible to get vaccinated	4 (2.4)	3 (4.3)	1 (3.2)	0 (0)	0.243
Other	12 (7.3)	4 (5.8)	2 (6.5)	6 (9.4)	0.795

Abbreviation: HCW, healthcare worker.

The majority of unvaccinated HCWs cited that they would get vaccinated if it was needed to secure a job (40.2%, 66/164), it was needed to protect their health (42.0%, 69/164) and the health of their family and friends (40.2%, 66/164), along with the provision of scientific or medical information (40.2%, 66/164) (Table 4).

**Table 4:** Key motivators for unvaccinated HCWs to get vaccinated.

Reasons, *n* (%)	Total (*n* = 164)	Healthcare workers/profession			*p* value
		Doctors (*n* = 69)	Nurses (*n* = 31)	Other HCWs (*n* = 64)	
The need to protect your health	69 (42.1)	23 (33.3)	20 (64.5)	26 (40.6)	**.013**
To protect the health of family/friends	66 (40.2)	25 (36.2)	13 (41.9)	28 (43.8)	.662
To protect health of co‐workers	46 (28.0)	16 (23.2)	11 (35.5)	19 (29.7)	.419
Encouragement to get vaccinated from friends and family	16 (9.8)	4 (5.8)	5 (16.1)	7 (10.9)	.222
Encouragement to get vaccinated from co‐workers	14 (8.5)	5 (7.2)	6 (19.4)	3 (4.7)	**.050**
Necessary to secure/maintain job	24 (14.6)	11 (15.9)	5 (16.1)	8 (12.5)	.826
More scientific or medical information is provided	66 (40.2)	28 (40.6)	13 (41.9)	25 (39.1)	.962
Have a friend/family contract COVID‐19	6 (3.7)	4 (5.8)	0 (0.0)	2 (3.1)	.566
Influential people in Tanzania get vaccinated	6 (3.7)	1 (1.4)	3 (9.7)	2 (3.1)	.136
Required to travel overseas	51 (31.1)	23 (33.3)	10 (32.3)	18 (28.1)	.801
Not sure	29 (17.7)	13 (18.8)	3 (9.7)	13 (20.3)	.421
Other	11 (3.0)	3 (4.3)	0 (0.0)	2 (3.1)	.842

Abbreviation: HCW, healthcare worker.

Regarding the information that would increase their willingness to get vaccinated, 70.7% (116/164) of unvaccinated HCWs revealed that they required more information about the vaccine's potential side effects. In comparison, 59.7% (98/164) desired more information on its efficacy. Additionally, 34.1% (56/164) expressed interest in knowing the vaccination status of COVID‐19‐related cases and deaths, as shown in Figure 5B.

### 3.5 Source of SARS‐CoV‐2 vaccine information

The role of HCWs as a potential source of information to promote SARS‐CoV‐2 vaccine uptake was investigated. The results revealed that vaccinated HCWs were more likely to provide advice (89.1%, 352/395) compared to unvaccinated HCWs (56.7%, 93/395) (*p* = .001). Most of HCWs (79.4%, 445/560) provided information and advice on SARS‐CoV‐2 vaccines. In comparison, 16.0% (90/560) of HCWs did not, and 4.4% (25/560) were unsure of providing COVID‐19 vaccine knowledge (Figure 5C). Notably, nurses were more likely to offer advice (93.4%, 141/151) compared to doctors (77.2%, 207/268) and other HCWs (68.8%, 97/141). These findings suggested that HCWs were perceived as reliable sources of information and could play a critical role in promoting vaccine uptake (Figure 5D).

## 4 DISCUSSION

According to research, the public trusts HCWs to respond to and combat COVID‐19. Furthermore, general public reflections on SARS‐CoV‐2 vaccination hesitancy have revealed that healthcare provider recommendations are associated with a lower SARS‐CoV‐2 vaccination hesitancy rate.^36^, ^37^ In this study, many HCWs reported offering knowledge or vaccination advice. However, the unvaccinated HCWs were less likely to do so than the vaccinated HCWs. This proves the hypothesis raised in the introduction section that HCWs are a potentially powerful influence on patient vaccination decisions and that vaccinated HCWs are likelier to recommend vaccination to others. Additionally, nurses were more likely than doctors or HCWs to advise or recommend SARS‐CoV‐2 vaccines to patients/families or relatives. However, a previous study showed that nurses were overall more negative towards SARS‐CoV‐2 vaccines.^38^ This finding suggests that HCWs, especially nurses, should be equipped with relevant information to enhance vaccine acceptance, given their comfort in discussing COVID‐19 and SARS‐CoV‐2 vaccine concerns with patients, friends and relatives.

The study showed that 70.5% of HCWs had received at least one dose of SARS‐CoV‐2 vaccines, indicating that a significant proportion of the HCWs in the country have taken steps to protect themselves from COVID‐19. It's notable that 12.8% of HCWs were unvaccinated but reported they would get vaccinated as soon as possible, which may be due to the relatively late initiation of vaccination in Tanzania,^39^ with the first doses administered in July 2021.^40^ This delay was primarily due to challenges in overcoming vaccine hesitancy, which had been exacerbated by the previous administration's antivaccination stance.^8^ Moreover, the data collection period of this study spanned from October to November 2022, during which time vaccination coverage in Tanzania remained relatively low, with 44.8% of individuals fully vaccinated.^41^

Nurses in Tanzania were more vaccinated than doctors and other HCWs. This is in contrast to studies conducted in other countries, citing doctors' greater awareness of the risks of COVID‐19.^42^, ^43^ This could be because nurses in Tanzania spend more time with patients than doctors, giving them a deeper understanding of the impact of COVID‐19.^44^

Notably, SARS‐CoV‐2 vaccination rates in Tanzania's central, southern, and lake zone regions are relatively high, possibly due to the targeted efforts of the Ministry of Health, which identified these areas as a high priority for vaccination because they were more affected by COVID‐19.^45^ Also, HCWs over 50 years have a double advantage as they fall in both priority groups and are prioritized for vaccination as they were deemed at greater risk of COVID‐19^46^; this might explain their higher vaccination rates.

The relationship between vaccination willingness and residency varies depending on the country.^47^, ^48^ Some prior research shows no significant association between residency and vaccination willingness.^49^ In this study, we found that rural respondents displayed a significantly higher willingness to be vaccinated against SARS‐CoV‐2. We can presume vaccine willingness in rural areas may be influenced by regular encounters with infectious diseases and the growing prevalence of vaccination practices. Additionally, the limited presence of vaccine‐related misconceptions in rural populations may also contribute to this trend.^47^ Notably, our study demonstrates a heightened level of vaccine coverage in rural regions, challenging existing research suggesting lower vaccination rates due to distribution challenges in these areas,^50^ suggesting the need for prompt research to identify and implement innovative solutions to address the disparities in coverage between urban and rural areas in Tanzania.

The current study showed that 81.4% of HCWs were willing to accept the SARS‐CoV‐2 vaccine. This shows a relatively higher willingness among HCWs in Tanzania than 80.9% by Konje et al.,^8^ possibly due to the role played by Tanzania's vaccine strategy and its implementing partners.^46^, ^51^ The strategy targeted frontline HCWS by developing micro‐plans and appropriate methods to reach all target groups, mobilizing targeted groups for vaccination, and implementing vaccination campaign activities by leveraging existing public awareness campaigns. The situation in Tanzania posed a unique context that may have initially seemed challenging. It's worth noting that Tanzania faced residual effects of the previous administration's influence, which included antivaccination sentiments and scepticism towards the COVID‐19 vaccine.^40^ Additionally, Tanzania embarked on its vaccination campaign relatively late compared to many other countries. However, it is indeed intriguing that despite these initial challenges, Tanzania's vaccination strategy ultimately proved effective, resulting in a high level of vaccination willingness among HCWs.^52^

In addition, the willingness to accept the SARS‐CoV‐2 vaccine was significantly associated with age, BMI, education, residency, and profession but not prior COVID‐19 diagnosis or co‐morbidities. High BMI, comorbidities, and prior diagnoses of COVID‐19 may influence an individual's perceived risk of COVID‐19 and, consequently, their inclination to accept vaccination. However, our study showed that comorbidities and prior diagnosis of COVID‐19 were not significantly associated with vaccine willingness among HCWs. This contrasts with other studies that reported higher vaccine willingness with HCWs with underlying diseases or conditions.^53^, ^54^ It may be because the influence of co‐morbidities and prior COVID‐19 diagnosis on vaccine willingness could vary by region, depending on factors such as local healthcare infrastructure and public health messaging.^55^, ^56^

HCWs with less than a high school education had a higher vaccination coverage rate, indicating that a larger proportion of them had received at least one dose of the COVID‐19 vaccine. It may be because people with low educational attainment know less about vaccines and worry less about safety.

Similar to other studies, HCWs with higher educational qualifications, such as a Master's degree or above, were more likely to accept the vaccine. The higher levels of education contributed to a better understanding and trust in scientific methods rather than beliefs in conspiracy theories.^7^, ^25^, ^57^ This finding suggests the need to provide targeted education and information to HCWs with a lower level of education to address their concerns and fears and enhance SARS‐CoV‐2 vaccine willingness.^58^

Nurses were more willing to take the SARS‐CoV‐2 vaccine than other HCWs. This greater willingness among nurses may be attributed to their increased exposure to SARS‐CoV‐2, their heightened perception of personal susceptibility to COVID‐19, brought by their frequent patient contact compared to other HCWs.^44^

The primary driver for willingness to be vaccinated was a collective responsibility, as HCWs pointed out that they got vaccinated because they are HCWs. HCWs in Tanzania understand the critical importance and their moral obligation to serve as role models. This was consistent with previous research from different countries on HCWs’ vaccine willingness.^28^, ^59^, ^60^ It's worth highlighting that in Tanzania, there is a unique emphasis on communal values. Tanzanian society places a significant emphasis on community and the collective well‐being of its members.^61^, ^62^ This cultural aspect is likely a significant contributor to the sense of shared responsibility among HCWs in Tanzania. Other reasons for their willingness observed in this study included government persuasion, media information, research, and the fear of increased COVID‐19 infections.

HCWs working in private health facilities were less likely than those working in government‐owned facilities to accept SARS‐CoV‐2 immunization. Based on our knowledge and the findings from the literature review, there were no vaccine mandates enforced for HCWs in government‐owned facilities.^63^ More research is needed to uncover the reasons for these disparities to inform tailor‐made vaccine promotion campaigns among the various cadres of HCWs based on their unique needs.

Vaccine hesitancy was reported by 62.5% of HCWs. Initially, COVID‐19 and SARS‐CoV‐2 vaccines were not accepted as a public health problem by the authority, and the community was assured of being safe without the vaccine.^39^, ^64^ This approach could have negatively affected the decision to uptake vaccination services among HCWs. Therefore, it is indicated that even if adequate vaccines were available, sufficiently high vaccination coverage might not be achieved when vaccine hesitancy among HCWs remains high.

Our results showed that the proportion of vaccine hesitancy to the SARS‐CoV‐2 vaccine was lower than that of a study (72%) conducted in the Democratic Republic of Congo^26^ and higher than that (41.0%) in a study in South Africa.^28^ Additionally, HCWs’ main reservations about the vaccine stemmed from concerns about its safety, efficacy, and side effects, similar to various studies.^7^, ^28^, ^65^ Additionally, these reasons were very similar to the main reasons given by HCWs in this study, which included potential side effects (68.9%), misinformation (11.0%), trust (12.2%), and the risk of contracting COVID‐19 from the vaccine (10.4%).

Vaccine hesitancy was distinct with age, with HCWs aged 18–30 years having the highest rate of vaccination refusal. This might be because they perceive themselves at lower risk of developing severe COVID‐19, partly explaining their vaccine hesitancy.^28^, ^66^ Female HCWs were more hesitant than men in this study. It has been discovered in other similar studies that females are less likely to receive a SARS‐CoV‐2 vaccine. It has been proposed that this is due to concerns about potential side effects such as infertility.^43^, ^67^ This was also a source of concern among unvaccinated HCWs in our study. When asked what additional information unvaccinated HCWs needed to be willing to be vaccinated, a portion of HCWs wanted to know about the effects of vaccines on their fertility.

There were some limitations to our study. Our study focused on the willingness, hesitancy, and coverage of SARS‐CoV‐2 vaccines among doctors, nurses, technicians, pharmacists, hospital administrators, and medical students in Tanzania. However, this specific focus may compromise the generalizability of the results. We used self‐identification without formal verification, highlighting limitations tied to this approach due to the potential inclusion of nonprofessionals via social media. Additionally, the study findings cannot be generalized as applicable to other HCW roles or other countries. Another limitation is that certain groups of HCWs, such as HCWs above 50 years (*n* = 24) and HCWs from the Zanzibar zone (*n* = 8), were underrepresented compared to others which may lead to biased results.

Additionally, while this study is unique in light of its broad coverage, it is also one of the few to have examined vaccine hesitancy and willingness among HCWs in Tanzania, a lack of external validity may still limit its generalizability across the country. It, therefore, calls for in‐depth studies of specific roles in different parts of the country to examine the other constructs that may affect vaccine hesitancy in a particular setting. Also, findings are from self‐reported responses. Self‐reported answers may be exaggerated and various biases, like social desirability bias.

Other limitations include the method of data collection and analysis. Most participants were contacted via electronic platforms, which may have excluded individuals who are not on those platforms or are not served by those platforms. Because of the electronic data deployed, it may have excluded individuals without access to such electronic tools. In addition, though measures were taken to ensure the enrollment of participants from all regions in Tanzania and professional groups, utilizing social media platforms did not guarantee this was so in all cases. Considering this challenge, future research can include site visits to conduct an interviewer‐administered paper version of the data collection tool. Using a convenient sampling technique limits the generalizability of the study findings and might create selection bias. It's a pity that we did not grade the degree of vaccine hesitation of the participants but treated it as a binary categorical variable, which limited the possibility of more analysis in the study.

## 5 CONCLUSION

Encouragingly, a large proportion of HCWs (70.5%) received the SARS‐CoV‐2 vaccine. The study identified factors contributing to vaccine hesitancy and concerns among unvaccinated HCWs. Some concerns included the short and long‐term effects, vaccine trust and safety, lack of information, and prevalent misinformation. There is an urgent need to create interventions to alleviate the fear and misunderstandings about the SARS‐CoV‐2 vaccines among health professionals to improve and sustain vaccine uptake among HCWs, who can be important in advocating for the vaccine among the general population. To increase vaccine acceptance among HCWs and the general population, targeted messaging is needed to deliver transparent information on vaccine safety, efficacy, and the development process.

## AUTHOR CONTRIBUTIONS

**Suzan Joseph Kessy**: Data curation; Investigation; Software; Visualization; Writing—original draft; Writing—review & editing. **Tingting Wei**: Data curation; Investigation; Methodology; Writing—original draft; Writing—review & editing. **Yiguo Zhou**: Investigation; Methodology; Software. **Wan‐Xue Zhang**: Data curation; Investigation; Writing—original draft. **Fadhlun M. Alwy Al‐Beity**: Conceptualization; Methodology; Project administration; Supervision. **Shan‐Shan Zhang**: Data curation; Formal analysis. **Juan Du**: Visualization; Writing—review & editing. **Fuqiang Cui**: Conceptualization; Methodology; Project administration; Supervision. **Qing‐Bin Lu**: Conceptualization; Methodology; Project administration; Supervision.

## CONFLICT OF INTEREST STATEMENT

The authors declare no conflicts of interest.

## Supporting information

Supporting information.

Supporting information.

## Data Availability

The original dataset presented in this study is publicly available, further inquiries can be directed to the corresponding authors.
