# Supplementary figures and images for: Vaccination willingness, vaccine hesitancy, and estimated coverage of SARS‐CoV‐2 vaccine among healthcare workers in Tanzania: A call for action
Source: Immun Inflamm Dis. 2023 Dec 26;11(12):e1126. doi: 10.1002/iid3.1126 (PMC10750438; doi:10.1002/iid3.1126)

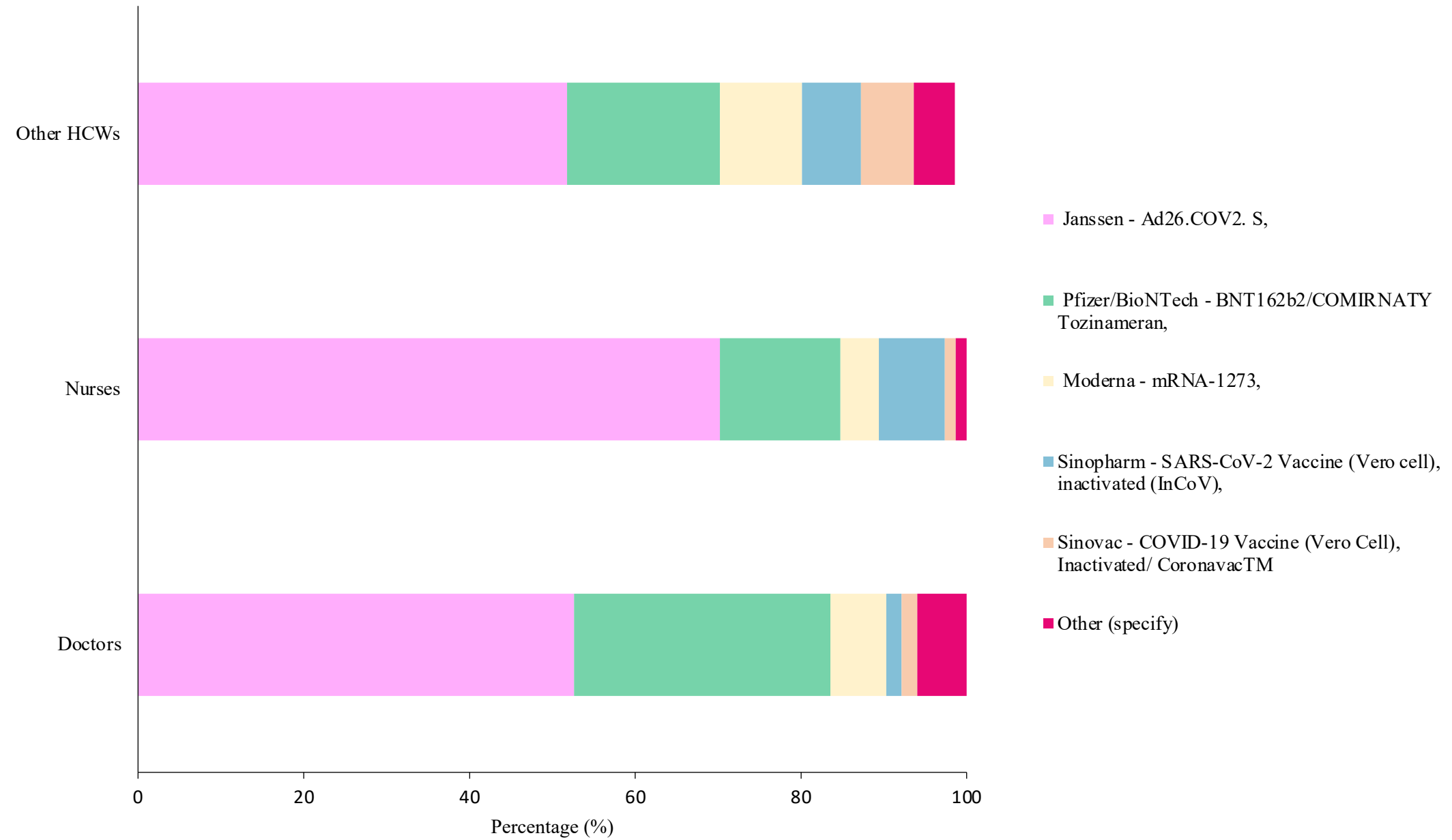

Supplement: Supplementary file 1 — Supporting information. [file IID3-11-e1126-s002.pdf]
