# Supplementary material for: Vaccination willingness, vaccine hesitancy, and estimated coverage of SARS‐CoV‐2 vaccine among healthcare workers in Tanzania: A call for action
Source: Immun Inflamm Dis. 2023 Dec 26;11(12):e1126. doi: 10.1002/iid3.1126 (PMC10750438; doi:10.1002/iid3.1126)
Supplement: Supplementary file 2 — Supporting information. [file IID3-11-e1126-s001.docx]

Supplement Table 1. COVID-19 Vaccine Hesitancy among Healthcare Workers in Tanzania (**Healthcare workers Questionnaire)**

Dear Participant,

I am inviting you to participate in this research by completing the following survey.

COVID-19 is an infectious disease that seriously endangers our health, and vaccination is considered one of the most effective means to prevent the spread of COVID-19. At present, the available vaccines in Tanzania are

1. Janssen - Ad26.COV2. S,
2. Pfizer/BioNTech - BNT162b2/COMIRNATY Tozinameran,
3. Moderna - mRNA-1273,
4. Sinopharm - SARS-CoV-2 Vaccine (Vero cell), inactivated (InCoV),
5. Sinovac - COVID-19 Vaccine (Vero Cell), Inactivated/ CoronavacTM

Limited take up of COVID-19 vaccines has been an ongoing issue hampering the response to the pandemic. This survey aims to provide a scientific basis for improving the vaccination coverage rate among Healthcare Workers.

This questionnaire will require approximately 10~15 minutes to complete. This is strictly a voluntary participation and to ensure privacy please do not include your name. There is no compensation and there is not any known risk for taking this survey. We have attached a consent form here, by selecting “Yes” button below you are indicating that you are sufficiently informed about the study and you want to participate voluntarily. Thank you for taking the time to assist me with my research.

**Are you sufficiently informed about the study and you want to participate voluntarily?**

|  | Part I: Sociodemographic information | |
| --- | --- | --- |
|  | Question | Answers |
| 1 | What is your sex? | 1. Male 2. Female |
| 2 | What is your age? | ……………….. (Years) |
| 2.1 | What is your current weight | ……… Kg |
| 2.3 | What is your height | ……… cm |
| 3 | What is your current role? | 1. Medical Doctor 2. Nurse 3. Medical technicians 4. Pharmacist 5. Hospital administrator 6. Medical students |
| 4 | What best describes your affiliation? | 1. Government Institution 2. Private Institution 3. Individual Business 4. Unemployed 5. Retiree 6. Other……………… |
| 4.1 | If you are unemployed (Only ask this question if someone selects Unemployed in the previous question), is this as a result of COVID-19? | 1. Not unemployed (Remove this from options 2. Working before and since COVID-19 (Remove this from options) 3. Unemployed before COVID-19 4. Unemployed because of COVID-19 5. Still at school 6. Won’t say |
| 4.2 | Do you currently work in any of the following locations? (Unemployed jump this) | 1. Community health center 2. Dispensary 3. District Hospital 4. Regional Hospital 5. Referral and specialized hospital 6. National Hospital 7. Other |
| 5 | What is the highest level of education you completed? | 1. Less than high school 2. High school or equivalent (e.g., college certificate) 3. College Diploma 4. Bachelor’s degree 5. Master’s degree or higher |
| 6 | Where do you currently reside? (Region) | (Multiple choice (31 options) |
| 6.1 | Do you currently reside in rural or urban area? | 1. Rural 2. Urban |
| 7 | Do you have any of the following conditions? (Select all that apply) | 1. Cancer 2. Chronic kidney disease 3. Chronic liver disease (such as chronic hepatitis, cirrhosis, etc.) 4. Chronic obstructive pulmonary disease (COPD) 5. Heart conditions, such as heart failure, coronary artery disease, or cardiomyopathies 6. Sickle cell disease 7. Type 2 diabetes mellitus 8. Hypertension 9. Hyperlipidemia 10. Immunocompromised due to solid organ transplant 11. Immune deficiencies (such as AIDS, etc.) 12. Current smoker 13. Pregnancy and Perinatal period 14. None of the above 15. Other underlying diseases or conditions |
| 8 | Are you currently covered by any form of health insurance or health plan? | 1. Yes 2. No 3. Not Sure |
| 9 | Have you been treated differently (poorly) by others during the COVID-19 pandemic because you are a healthcare worker? | 1. Yes 2. No 3. Not sure |
|  | PART II: COVID-19 | |
| 1. | To your knowledge, do you have or have you had COVID-19? | 1. Yes 2. No 3. I don’t know |
| 2. | If “Yes,” describe the level of care you received, or are receiving: | 1. Did not seek medical care 2. Used herbal medicines (ginger, lemon, COVIDOL etc.) 3. Received medical care but was not hospitalized 4. Was hospitalized |
| 3. | What measures did you take for COVID-19 mitigation? (select all that apply) | 1. Social distancing 2. Hand hygiene 3. Dining attitudes, beliefs, and behaviors 4. Face mask wearing 5. Steam therapy 6. Prayers |
| 4 | Do you personally know anyone in your family, group of friends, or community networks who became seriously ill or died as a result of COVID-19? | 1. Yes 2. No |
|  | PART III: Vaccination status | |
| 1. | Have you received a COVID-19 vaccine? | 1. Yes 2. No 3. Not sure |
| 1.1 | If “Yes” which of these vaccines, did you receive? (Select all that apply) | 1. Janssen - Ad26.COV2. S 2. Pfizer/BioNTech - BNT162b2/COMIRNATY Tozinameran 3. Moderna - mRNA-1273 4. Sinopharm - SARS-CoV-2 Vaccine (Vero cell), inactivated (InCoV) 5. Sinovac - COVID-19 Vaccine (Vero Cell), Inactivated/CoronavacTM |
| 1.2 | If “Yes”, did you choose your vaccination type, or did you just take what was available at the time? | 1. Yes, Chose my preferred vaccine 2. No, I took what was available |
| 1.3 | If ‘Yes’, how many doses does your vaccine require? | 1. One dose 2. Two doses 3. I don’t know |
| 1.4 | If two doses, have you completed your second dose? | 1. Yes 2. No |
| 1.5 | If ‘No’, what are the reasons for not getting the second dose? | 1. I got vaccinated recently, I will get my second dose on time 2. I don’t know where to go to get the second dose 3. I have a medical reason (e.g., I got a severe allergy my last dose). 4. It is difficult to find or make an appointment. 5. Other (please specify)   _______________________________  _______________________________ |
| 1.6 | I think my decision on COVID-19 vaccination was right. | 1. Strongly disagree 2. Disagree 3. Neutral 4. Agree 5. Strongly agree |
| 1.7 | (Remove this question for not vaccinated people)  What influenced your decision to get vaccinated? | 1. I was satisfied with the information I saw in the media (radio, TV, social media) 2. My spiritual/religious leader advised me 3. Persuaded by government/public health authorities 4. Noticed that a personality (social influencer/ public or otherwise) took it 5. Felt more comfortable because several people took it 6. I was forced by my employer/family members/didn’t have a choice 7. I did more research on COVID-19 vaccines 8. I am a healthcare worker I had to lead by example 9. Frightened by the increased infections/deaths 10. My circumstances changed (such as being pregnant or other medical or personal factor) 11. Offered the vaccine with which I was more comfortable 12. I was influenced by my colleagues 13. Consulted family and friends 14. Other________________________   _____________________________ |
| 1.8 | Were you initially hesitant to take the vaccine? | 1. Yes 2. No |
| 1.9 | If you were hesitant, please explain why you were initially hesitant | 1. I did not qualify to receive it before 2. I did not like any of the vaccine options available to me 3. I had religious grounds for not taking it 4. I had a medical condition that barred me from taking it 5. I did not trust the government 6. I did not trust medical authorities 7. I did not trust the vaccines (not safe, developed too quickly, do not know what is in it) 8. I was not sure about long-term side effects of the vaccine 9. Other ____________________   __________________________ |
| 1.10 | If you were hesitant, what changed your mind? (Select option that best apply) | 1. I was satisfied with the information I saw in the media (radio, TV, social media) 2. My spiritual/religious leader advised me 3. Persuaded by government/public health authorities 4. Noticed that a personality (social influencer/ public or otherwise) took it 5. Felt more comfortable because several people took it 6. I was forced by my employer/family members/didn’t have a choice 7. I did more research on COVID-19 vaccines 8. I am a healthcare worker I had to lead by example 9. Frightened by the increased infections/deaths 10. My circumstances changed (such as being pregnant or other medical or personal factor) 11. Offered the vaccine with which I was more comfortable 12. I was influenced by my colleagues 13. Consulted family and friends 14. Other________________________   _____________________________ |
| 1.11 | How likely are you to recommend getting the COVID-19 vaccine to others? | 1. Not at all likely 2. Somewhat likely 3. Extremely likely |
| 1.12 | How easy do you think it will be to get a COVID-19 vaccine? | 1. Very easy 2. Somewhat easy 3. Somewhat difficult 4. Very difficult 5. Not sure |
| 1.13 | If a COVID-19 vaccine were available to you, would you get it? | 1. Yes, would get it as soon as possible 2. Yes, but plan to wait to get it 3. No 4. Not sure |
| 1.14 | Which of these reasons best explains why you are unvaccinated at this time? (Please select all that apply) | 1. I am not sure about short or long-term side effects of the vaccine 2. There is a risk of contracting COVID-19 3. I will not take it on religious grounds, 4. Beliefs, culture and traditions do not allow me to be vaccinated 5. I believe it is a choice and I choose not to 6. No reason really, I just won’t take it 7. It is not mandatory for my work 8. Have heard (positive or negative) news from media (TV, Radios, and social media) 9. I am too busy and too much time is spent on the inoculation process 10. There are no rewards after vaccination 11. Recommendations from people around me 12. People around me are vaccinated and their reaction after vaccination 13. Attitudes and techniques of vaccinators 14. I do not trust the government/medical authorities here I do not trust the vaccine (not safe, developed too quickly, do not know what is in it) 15. Previous experience or side effects of vaccination 16. I don’t like any of the vaccine options available to me 17. I don’t know where to go to get vaccinated. 18. I’m not eligible to get a COVID-19 vaccine. I have a medical reason that makes me ineligible to get vaccinated 19. Other_ |
| 1.15 | Has your view on the COVID-19 vaccination always been the same or has it changed over time? | 1. No change- I still am not going to take it 2. I am now more inclined towards taking it 3. I am now less inclined towards taking it |
| 1.16 | What would motivate you to get vaccinated? (Select all that apply.) | 1. The need to protect your health 2. To protect health of family/friends 3. To protect health of co-workers 4. Encouragement to get vaccinated from friends and family 5. Encouragement to get vaccinated from coworkers 6. Necessary to secure/maintain job 7. More scientific or medical information is provided 8. Have a friend/ family contract COVID-19 9. Influential people in Tanzania get vaccinated 10. Required to travel overseas 11. Not sure   Other ___________________________ |
| 1.17 | Specifically, what type of information on the COVID-19 Vaccine would help you be more inclined to take it? (Please select all that apply) | 1. The side effects of the vaccine 2. How effective the vaccines are 3. The numbers of people who got sick/died and their vaccination status 4. The different types of vaccines available 5. Location of vaccination sites 6. The position of my Church or Religious leader 7. The impact of the vaccine on my sexual health and fertility. |
| 1.18 | If the following people recommend that you get vaccinated or not, who are you more likely to listen to? | 1. Medical workers 2. Family and friends 3. Government workers 4. News media |
| PART IV: Counseling Behavior Survey | | |
| 1 | How comfortable do you feel addressing patient concerns about the COVID-19 vaccine (e.g., concerns about side effects)? | 1. Very comfortable 2. Somewhat comfortable 3. Neutral 4. Somewhat uncomfortable 5. Very uncomfortable |
| 2 | Do you think most of the people at your work or school or community will get a COVID-19 vaccine, if it is recommended for them? | 1. Yes 2. No 3. Not sure |
| 3 | Do you offer knowledge or vaccination advice? | 1. Yes 2. No 3. Not sure |
|  | PART V: Vaccine Preference | |
| 1 | Which vaccine would you prefer to choose for vaccination or future booster vaccinations for COVID-19 vaccines? | 1. Janssen - Ad26.COV2. S, 2. Pfizer/BioNTech - BNT162b2/COMIRNATY Tozinameran, 3. Moderna - mRNA-1273, 4. Sinopharm - SARS-CoV-2 Vaccine (Vero cell), inactivated (InCoV), 5. Sinovac - COVID-19 Vaccine (Vero Cell), Inactivated/ CoronavacTM 6. Other (specify) |
| 2 | What properties are you more concerned about the COVID-19 vaccine? Please select all that apply____ | 1. Vaccine types (e.g., inactivated, non-inactivated vaccines) 2. Production area (e.g., import, domestic production) 3. Security 4. Immune effect 5. Immune persistence 6. Vaccination rewards 7. Vaccination sites (e.g., community vaccination, self-vaccination in the hospital) |
|  | PART VI: Vaccination Mandate | |
| 1 | Would you support the imposition of COVID-19 vaccine mandates in respect of: (select Yes or No for each statement)   \| Population \| Yes \| No \| Don’t know \| \| --- \| --- \| --- \| --- \| \| Arriving visitors \|  \|  \|  \| \| Frontline medical/Elder care workers \|  \|  \|  \| \| Public servants \|  \|  \|  \| \| Workers in the Hotel/Tourism sector \|  \|  \|  \| \| Taxi/Minibus drivers and conductors \|  \|  \|  \| \| Secondary/Tertiary school children \|  \|  \|  \| \| Primary school children \|  \|  \|  \| | |
|  | PART VII: COVID-19 Information | |
| 1 | Have you seen or heard any information about COVID-19 vaccines (e.g., on the news, on social media, or from friends and family) that you could not determine were true or false? | 1. Yes 2. No 3. Not sure |
| 2 | Do you find the information conveyed by popular personalities (local and international) on social media helpful in terms of forming your own opinions on COVID-19 related matters? | 1. Yes, very helpful 2. Somewhat helpful 3. Unsure 4. Won’t say |
| 3 | How do you feel about the amount of information on COVID-19 vaccines that you are getting? | 1. I’m not getting enough information. 2. I’m getting enough information. 3. I’m getting too much information. |
| 4 | Do you know where to get accurate, timely information about COVID-19 vaccines? | 1. Yes 2. No 3. Not sure |
| 5 | Select your top 3 most trusted sources of information about COVID-19 vaccines: | 1. Regional and International Health Organizations (Africa CDC, WHO etc.) 2. Ministry of Health, Tanzania 3. National Health Institutes (NIMR, TMDA etc.) 4. Employer 5. Family and friends 6. Health insurers 7. Local health officials News sources (e.g., television, internet, and radio) 8. Healthcare Workers 9. Religious leader(s) 10. Online publishers of medical information (such as WebMD or Mayo Clinic) 11. Social media (such as Facebook, Twitter, Instagram, WhatsApp, LinkedIn, or TikTok) 12. Other |

Supplement Table 2. Classification of Zones

| Zone | Regions |
| --- | --- |
| Northern Zone | Arusha |
|  | Kilimanjaro |
|  | Manyara |
|  | Tanga |
| Central Zone | Dodoma |
|  | Morogoro |
|  | Singida |
|  | Tabora |
| Lake Zone | Geita |
|  | Kagera |
|  | Kigoma |
|  | Mara |
|  | Mwanza |
|  | Shinyanga |
|  | Simiyu |
| Southern Zone | Ruvuma |
|  | Pwani |
|  | Lindi |
|  | Mtwara |
| Southern Highlands Zone | Katavi |
|  | Mbeya |
|  | Songwe |
|  | Njombe |
|  | Rukwa |
|  | Iringa |
| Zanzibar | Unguja North |
|  | Unguja South |
|  | Unguja urban west |
|  | Pemba North |
|  | Pemba South |
| Dar es salaam | Dar es salaam |

Supplement Table 3. Characteristics of healthcare workers by six professions

| Characteristics, n (%) | Total (N=560) | Healthcare Workers/Profession | | | | | |
| --- | --- | --- | --- | --- | --- | --- | --- |
|  |  | Doctors (n=268) | Nurse (n=151) | Medical technicians (n=45) | Pharmacist (n=24) | Hospital administrator (n=30) | Medical students (n=42) |
| **Sex** |  |  |  |  |  |  |  |
| Male | 322 (57.5) | 166 (61.9) | 74 (49.0) | 30 (66.7) | 12 (50.0) | 16 (53.3) | 26 (57.1) |
| Female | 238 (42.5) | 102 (38.1) | 77 (51.0) | 15 (33.3) | 12 (50.0) | 14 (46.7) | 18 (42.9) |
| **Age group** |  |  |  |  |  |  |  |
| Under 30 years | 242 (43.2) | 63 (23.5) | 94 (62.3) | 24 (53.3) | 16 (66.7) | 14 (46.7) | 31 (73.8) |
| 30-49 years | 294 (52.9) | 190 (70.9) | 53 (35.1) | 20 (44.4) | 6 (25.0) | 16 (53.3) | 11 (26.2) |
| Above 50 years | 24 (3.9) | 15 (5.6) | 4 (2.6) | 1 (2.3) | 2 (8.3) | 0 (0) | 0 (0) |
| **BMI** |  |  |  |  |  |  |  |
| $\leq$18.5 | 23 (4.1) | 9 (3.4) | 7 (4.6) | 1 (2.2) | 2 (8.3) | 2 (6.7) | 2 (4.8) |
| 18.5–24.9 | 224 (40.0) | 93 (34.7) | 67 (44.4) | 20 (44.4) | 9 (37.5) | 10 (33.3) | 25 (59.5) |
| 25–29.9 | 198 (35.4) | 111 (41.4) | 48 (31.8) | 9 (20.0) | 8 (33.3) | 11 (36.7) | 11 (26.2) |
| >30 | 115 (20.5) | 55 (20.5) | 29 (19.2) | 15 (33.3) | 5 (20.8) | 7 (23.3) | 4 (9.5) |
| **Insurance** |  |  |  |  |  |  |  |
| Yes | 427 (76.3) | 224 (83.6) | 107 (70.9) | 31 (68.9) | 10 (41.7) | 21 (70.0) | 34 (81.0) |
| No | 125 (22.3) | 41 (15.3) | 42 (27.8) | 14 (31.1) | 14 (58.3) | 7 (23.3) | 7 (16.7) |
| Not sure | 8 (1.4) | 3 (1.1) | 2 (1.3) | 0 (0) | 0 (0) | 2 (6.7) | 1 (2.4) |
| **Education** |  |  |  |  |  |  |  |
| Never | 1 (0.2) | 0 (0) | 0 (0) | 0 (0) | 0 (0) | 0 (0) | 0 (0) |
| Less than high school | 8 (1.4) | 0 (0) | 6 (4.0) | 1 (2.2) | 0 (0) | 0 (0) | 1 (2.4) |
| High school or equivalent (e.g., college certificate) | 33 (5.9) | 1 (0.4) | 14 (9.3) | 2 (4.4) | 1 (4.2) | 2 (6.7) | 13 (31.0) |
| College Diploma | 122 (21.8) | 13 (4.9) | 79 (52.3) | 15 (33.3) | 4 (16.7) | 3 (10.0) | 8 (19.0) |
| Bachelor’s degree | 260 (46.4) | 148 (55.2) | 42 (27.8) | 17 (37.8) | 18 (75.0) | 19 (63.3) | 16 (38.1) |
| Master’s degree or higher | 136 (24.3) | 106 (39.6) | 10 (6.6) | 10 (22.2) | 1 (4.2) | 6 (20.0) | 3 (7.1) |
| **Residency** |  |  |  |  |  |  |  |
| Urban | 458 (81.8) | 230 (85.8) | 116 (76.8) | 32 (71.1) | 23 (95.8) | 22 (73.3) | 35 (83.3) |
| Rural | 102 (18.2) | 38 (14.2) | 35 (23.6) | 13 (22.9) | 1 (4.2) | 8 (26.7) | 7 (16.7) |
| **Residency (Zone)**^[[1]](#footnote-1)^ |  |  |  |  |  |  |  |
| Northern Zone | 75 (13.4) | 32 (42.7) | 27(36.0) | 7 (15.6) | 2 (8.3) | 2 (6.7) | 5 (11.9) |
| Central Zone | 42(7.5) | 24(57.1) | (31.0) | 2 (4.4) | 0 (0) | 1 (3.3) | 2 (4.8) |
| Lake Zone | 120 (21.4) | 46(38.3) | 51(42.5) | 10 (22.2) | 5 (20.8) | 5 (16.7) | 3 (7.1) |
| Southern Zone | 33 (5.9) | 14(42.4) | 16(48.5) | 8 (17.8) | 0 (0) | 1 (3.3) | 6 (14.3) |
| Southern highlands | 41 (7.3) | 15(36.6) | 11(26.8) | 1 (2.2) | 0 (0) | 2 (6.7) | 0 (0) |
| Zanzibar Zone | 8 (1.4) | 5(62.5) | 0 (0) | 0 (0) | 1 (4.2) | 1 (3.3) | 1 ( 2.4) |
| Dar es Salaam Zone | 241 (43.0) | 132(54.5) | 33(13.7) | 17 (37.8) | 16 (66.7) | 18 (60.0) | 25 (59.5) |
| **Healthcare Setting** |  |  |  |  |  |  |  |
| Government Institution | 309 (55.2) | 170 (63.4) | 78 (51.7) | 26 (57.8) | 6 (25.0) | 16 (53.3) | 13 (31.0) |
| Private Institution | 167 (29.8) | 71 (26.5) | 50 (33.1) | 14 (31.1) | 12 (50.0) | 10 (33.3) | 10 (23.8) |
| Individual Business | 11 (2.0) | 6 (2.2) | 2 (1.3) | 0 (0) | 2 (8.3) | 0 (0) | 1 (2.4) |
| Unemployed | 52 (9.3) | 9 (3.4) | 17 (11.3) | 5 (11.1) | 3 (12.5) | 4 (13.3) | 14 (33.3) |
| Retiree | 5 (0.9) | 3 (1.1) | 0 (0) | 0 (0) | 1 (4.2) | 0 (0) | 1 (2.4) |
| Other | 16 (2.9) | 9 (3.4) | 4 (2.6) | 0 (0) | 0 (0) | 0 (0) | 3 (7.1) |
| **Healthcare Setting** |  |  |  |  |  |  |  |
| Community health centre | 76 (13.6) | 33 (43.4) | 23 (30.3) | 3 (3.9) | 6 (7.9) | 5 (6.6) | 6 (7.9) |
| Dispensary | 31 (5.5) | 10 (3.9) | 12 (9.0) | 6 (13.3) | 1 (4.2) | 2 (6.7) | 0 (0) |
| District Hospital | 77 (13.8) | 28 (10.8) | 38 (28.4) | 7 (15.6) | 0 (0) | 1 (3.3) | 3 (7.1) |
| Regional Hospital | 59 (10.5) | 35 (13.5) | 20 (14.9) | 2 (4.4) | 1 (4.2) | 1 (3.3) | 0 (0) |
| Referral and specialized hospital | 96 (17.1) | 63 (24.3) | 15 (11.2) | 6 (13.3) | 3 (12.5) | 4 (13.3) | 5 (11.9) |
| National Hospital | 51 (9.1) | 31 (12.0) | 10 (7.5) | 1 (2.2) | 0 (0) | 1 (3.3) | 8 (19.0) |
| Other | 118 (21.1) | 59 (22.8) | 16 (11.9) | 15 (33.3) | 10 (41.7) | 12 (40.0) | 6 (14.3) |
| Unemployed | 52 (9.3) | 9 (3.4) | 17 (11.3) | 5 (11.1) | 3 (12.5) | 4 (13.3) | 14 (33.3) |
| **Chronic Disease** |  |  |  |  |  |  |  |
| Yes | 79 (13.6) | 38 (14.2) | 19 (12.6) | 6 (13.3) | 6 (25.0) | 7 (23.3) | 3 (7.1) |
| No | 481 (86.4) | 230 (85.8) | 132 (87.4) | 39 (86.7) | 18 (75.0) | 23 (76.7) | 39 (92.9) |
| **History of COVID-19 Infection** |  |  |  |  |  |  |  |
| Yes | 176 (31.4) | 105 (39.2) | 30 (19.9) | 10 (22.2) | 5 (20.8) | 10 (33.3) | 16 (38.1) |
| No | 323 (57.7) | 128 (47.8) | 111 (73.5) | 30 (66.7) | 17 (70.8) | 15 (50.0) | 22 (52.4) |
| I don’t know | 61 (10.9) | 35 (13.1) | 10 (6.6) | 5 (11.1) | 2 (8.3) | 5 (16.7) | 4 (9.5) |

Supplement Table 4. Factors associated with HCWs' complete vaccination.

| Variables, n (%) | Complete vaccination (n=387) | Without complete vaccination (n=172) | P value |
| --- | --- | --- | --- |
| **Sex** |  |  | 0.112 |
| Male | 232 (72.0) | 90 (28.0) |  |
| Female | 155 (65.4) | 82 (34.6) |  |
| **Age** |  |  | <0.001 |
| <30 years | 142 (58.7) | 100 (41.3) |  |
| 30-49 years | 223 (76.1) | 70 (23.9) |  |
| ≥50 years | 22 (91.7) | 2 (8.3) |  |
| **BMI, Kg/m^2^** |  |  |  |
| ≤18.5 | 16 (69.6) | 7 (30.4) | 0.023 |
| 18.5–24.9 | 140 (62.5) | 84 (37.5) |  |
| 25–29.9 | 142 (71.7) | 56 (28.3) |  |
| ≥30 | 89 (78.1) | 25 (21.9) |  |
| **Education** |  |  | <0.001 |
| Less than high school | 7 (87.5) | 1 (12.5) |  |
| High school or equivalent | 20 (60.6) | 13 (39.4) |  |
| College Diploma | 96 (78.7) | 26 (21.3) |  |
| Bachelor’s degree | 154 (59.2) | 106 (40.8) |  |
| Master’s degree or higher | 110 (80.9) | 26 (19.1) |  |
| **Zones** |  |  | <0.001 |
| Central Zone | 37 (88.1) | 5 (11.9) |  |
| Dar es salaam | 138 (57.3) | 103 (42.7) |  |
| Lake Zone | 95 (79.2) | 25 (20.8) |  |
| Northern Zone | 53 (70.7) | 22 (29.3) |  |
| Southern Highlands Zone | 32 (78.0) | 9 (22.0) |  |
| Southern Zone | 28 (84.8) | 5 (15.2) |  |
| Zanzibar | 4 (57.1) | 3 (42.9) |  |
| **Residency** |  |  | <0.001 |
| Rural | 88 (86.3) | 14 (13.7) |  |
| Urban | 299 (65.4) | 158 (34.6) |  |
| **Affiliation** |  |  | 0.046 |
| Government Institution | 229 (74.1) | 80 (25.9) |  |
| Private Institution | 110 (65.9) | 57 (34.1) |  |
| Individual Business | 6 (54.5) | 5 (45.5) |  |
| Unemployed | 28 (54.9) | 23 (45.1) |  |
| Retiree | 3 (60.0) | 2 (40.0) |  |
| Other | 11 (68.8) | 5 (31.3) |  |
| **Workplace** |  |  | **0.024** |
| Community health center | 60 (78.9) | 16 (21.1) |  |
| Dispensary | 25 (80.6) | 6 (19.4) |  |
| District Hospital | 61 (79.2) | 16 (20.8) |  |
| Regional Hospital | 42 (71.2) | 17 (28.8) |  |
| Referral and specialized hospital | 57 (59.4) | 39 (40.6) |  |
| National Hospital | 30 (58.8) | 21 (41.2) |  |
| Other | 84 (71.2) | 34 (28.8) |  |
| Unemployed | 13 (68.4) | 6 (31.6) |  |
| **Chronic diseases** |  |  | 0.692 |
| Yes | 56 (71.8) | 22 (28.2) |  |
| No | 331 (68.8) | 150 (31.2) |  |
| **Insurance** |  |  | 0.294 |
| Yes | 301 (70.5) | 126 (29.5) |  |
| No | 82 (66.1) | 42 (33.9) |  |
| Not sure | 4 (50.0) | 4 (50.0) |  |
| **Role** |  |  | <0.001 |
| Doctor | 193 (72.0) | 75 (28.0) |  |
| Nurse | 118 (78.1) | 33 (21.9) |  |
| Others | 76 (54.3) | 64 (45.7) |  |

Supplement Table 5. Factors associated with HCWs' vaccination

| Variables, n (%) | Vaccination (n=395) | Without vaccination (n=164) | P value |
| --- | --- | --- | --- |
| **Sex** |  |  | 0.134 |
| Male | 236 (73.3) | 86 (26.7) |  |
| Female | 159 (66.8) | 78 (32.9) |  |
| **Age** |  |  | **<0.001** |
| <30 years | 146 (60.3) | 96 (39.7) |  |
| 30-49 years | 226 (76.9) | 67 (22.9) |  |
| ≥50 years | 23 (95.8) | 1 (4.2) |  |
| **BMI, Kg/m^2^** |  |  | **0.015** |
| ≤18.5 | 17 (73.9) | 6 (26.1) |  |
| 18.5–24.9 | 142 (63.4) | 82 (36.5) |  |
| 25–29.9 | 146 (73.7) | 52 (26.3) |  |
| ≥30 | 90 (78.3) | 24 (21.1) |  |
| **Education** |  |  | **<0.001** |
| Less than high school | 7 (87.5) | 1 (12.5) |  |
| High school or equivalent | 20 (60.6) | 13 (39.4) |  |
| College Diploma | 98 (80.3) | 24 (19.7) |  |
| Bachelor’s degree | 157 (60.4) | 103 (39.6) |  |
| Master’s degree or higher | 113 (83.1) | 23 (16.9) |  |
| **Zones** |  |  | **<0.001** |
| Central Zone | 37 (88.1) | 5 (11.9) |  |
| Dar es salaam | 144 (59.8) | 97 (40.2) |  |
| Lake Zone | 96 (80.0) | 24 (20.0) |  |
| Northern Zone | 54 (72.0) | 21 (28.0) |  |
| Southern Highlands Zone | 32 (78.0) | 9 (22.0) |  |
| Southern Zone | 28 (84.8) | 5 (15.2) |  |
| Zanzibar | 4 (57.1) | 3 (42.9) |  |
| **Residency** |  |  | **<0.001** |
| Rural | 88 (86.3) | 14 (13.7) |  |
| Urban | 307 (67.2) | 150 (32.8) |  |
| **Affiliation** |  |  | 0.088 |
| Government Institution | 232 (75.1) | 77 (24.9) |  |
| Private Institution | 112 (67.1) | 55 (32.9) |  |
| Individual Business | 7 (63.6) | 4 (36.4) |  |
| Unemployed | 29 (56.9) | 22 (43.1) |  |
| Retiree | 4 (80.0) | 1 (20.0) |  |
| Other | 11 (68.8) | 5 (31.2) |  |
| **Workplace** |  |  | **0.007** |
| Community health center | 60 (78.9) | 16 (21.1) |  |
| Dispensary | 25 (80.6) | 6 (19.4) |  |
| District Hospital | 62 (80.5) | 15 (19.5) |  |
| Regional Hospital | 42 (71.2) | 17 (28.8) |  |
| Referral and specialized hospital | 60 (62.5) | 36 (37.5 |  |
| National Hospital | 30 (58.8) | 21 (41.2) |  |
| Other | 87 (73.7) | 31 (26.3) |  |
| Unemployed | 29 (56.9) | 22 (43.1) |  |
| **Chronic diseases** |  |  | 0.523 |
| Yes | 58 (74.4) | 20 (25.6) |  |
| No | 337 (70.1) | 144 (29.9) |  |
| **Insurance** |  |  | 0.269 |
| Yes | 307 (71.9) | 120 (28.1) |  |
| No | 84 (67.7) | 40 (32.3) |  |
| Not sure | 4 (50.0) | 4 (50.0) |  |
| **Role** |  |  | **<0.001** |
| Doctor | 199 (74.3) | 69 (25.7) |  |
| Nurse | 120 (79.5) | 31 (20.5) |  |
| Others | 76 (54.3) | 64 (45.7) |  |

Supplement Table 6. Logistic regression of HCWs' vaccine HCWs' vaccination

|  | Estimate | Std.Error | Z value | P value | OR (95%CI) |
| --- | --- | --- | --- | --- | --- |
| **Role** |  |  |  |  |  |
| Doctor | - | - | - | - | ref |
| Nurse | 0.070 | 0.318 | 0.219 | 0.827 | 1.072 (0.577~2.016) |
| Others | -0.807 | 0.284 | -2.836 | **0.005** | 0.446 (0.255~0.778) |
| **Sex** |  |  |  |  |  |
| Male | - | - | - | - | ref |
| Female | -0.167 | 0.227 | -0.737 | 0.461 | 0.846 (0.542~1.322) |
| **Age** |  |  |  |  |  |
| <30 years | - | - | - | - | ref |
| 30-49 years | 0.643 | 0.270 | 2.384 | **0.017** | 1.902 (1.125~3.244) |
| ≥50 years | 2.417 | 1.176 | 2.055 | **0.040** | 11.212 (1.756~271.326) |
| **BMI, Kg/m^2^** |  |  |  |  |  |
| ≤18.5 | - | - | - | - | ref |
| 18.5–24.9 | -0.490 | 0.546 | -0.896 | 0.370 | 0.613 (0.196~1.718) |
| 25–29.9 | -0.289 | 0.563 | -0.513 | 0.608 | 0.749 (0.233~2.176) |
| ≥30 | -0.022 | 0.587 | -0.037 | 0.971 | 0.979 (0.292~2.996) |
| **Education** |  |  |  |  |  |
| Less than high school | - | - | - | - | ref |
| High school or equivalent | -0.946 | 1.233 | -0.767 | 0.443 | 0.388 (0.017~3.356) |
| College Diploma | -0.453 | 1.192 | -0.381 | 0.704 | 0.635 (0.030~4.878) |
| Bachelor’s degree | -1.374 | 1.186 | -1.159 | 0.247 | 0.253 (0.012~1.923) |
| Master’s degree or higher | -0.436 | 1.226 | -0.356 | 0.722 | 0.647 (0.029~5.407) |
| **Zones** |  |  |  |  |  |
| Central Zone | - | - | - | - | ref |
| Dar es salaam | -0.801 | 0.565 | -1.417 | 0.156 | 0.449 (0.134~1.273) |
| Lake Zone | -0.081 | 0.581 | -0.139 | 0.890 | 0.923 (0.269~2.721) |
| Northern Zone | -0.539 | 0.592 | -0.910 | 0.363 | 0.583 (0.167~1.767) |
| Southern Highlands Zone | 0.074 | 0.667 | 0.112 | 0.911 | 1.077 (0.274~3.897) |
| Southern Zone | -0.167 | 0.731 | -0.229 | 0.819 | 0.846 (0.196~3.636) |
| Zanzibar | -1.579 | 1.040 | -1.517 | 0.129 | 0.206 (0.026~1.653) |
| **Residency** |  |  |  |  |  |
| Rural | - | - | - | - | ref |
| Urban | -0.618 | 0.383 | -1.614 | 0.107 | 0.539 (0.246~1.115) |
| **Affiliation** |  |  |  |  |  |
| Government Institution | - | - | - | - | ref |
| Private Institution | -0.638 | 0.274 | -2.324 | **0.020** | 0.528 (0.307~0.903) |
| Individual Business | -0.547 | 0.757 | -0.723 | 0.470 | 0.578 (0.131~2.714) |
| Unemployed | -0.884 | 0.507 | -1.744 | 0.081 | 0.413 (0.151~1.107) |
| Retiree | -1.199 | 1.598 | -0.750 | 0.453 | 0.302 (0.009~9.567) |
| Other | -0.790 | 0.637 | -1.240 | 0.215 | 0.454 (0.133~1.689) |
| **Workplace** |  |  |  |  |  |
| Community health centre | - | - | - | - | ref |
| Dispensary | -0.407 | 0.641 | -0.634 | 0.526 | 0.666 (0.193~2.445) |
| District Hospital | -0.085 | 0.457 | -0.187 | 0.852 | 0.918 (0.372~2.252) |
| Regional Hospital | -0.548 | 0.473 | -1.157 | 0.247 | 0.578 (0.226~1.459) |
| Referral and specialized hospital | -0.928 | 0.423 | -2.192 | **0.028** | 0.396 (0.169~0.894) |
| National Hospital | -1.006 | 0.505 | -1.991 | **0.046** | 0.366 (0.134~0.975) |
| Other | 0.063 | 0.422 | 0.150 | 0.881 | 1.065 (0.461~2.428) |
| **Chronic diseases** |  |  |  |  |  |
| Yes | - | - | - | - | ref |
| No | 0.169 | 0.327 | 0.517 | 0.605 | 1.184 (0.631~2.287) |
| **Insurance** |  |  |  |  |  |
| Yes | - | - | - | - | ref |
| No | 0.352 | 0.282 | 1.249 | 0.212 | 1.422 (0.823~2.490) |
| Not sure | -0.339 | 0.803 | -0.422 | 0.673 | 0.713 (0.141~3.578) |

Supplement Table 7. Factors associated with HCWs' vaccine willingness.

| Variables, n (%) | Willing (n=456) | Unwilling (n=103) | P value |
| --- | --- | --- | --- |
| **Sex** |  |  | 0.286 |
| Male | 268 (83.2) | 54 (16.8) |  |
| Female | 188 (79.0) | 49 (21.0) |  |
| **Age** |  |  | **0.006** |
| <30 years | 184 (76.0) | 58 (24.0) |  |
| 30-49 years | 249 (85.0) | 44 (15.0) |  |
| ≥50 years | 23 (95.8) | 1 (4.2) |  |
| **BMI** |  |  | **0.002** |
| ≤18.5 | 20 (87.0) | 3 (13.0) |  |
| 18.5–24.9 | 167 (74.6) | 57 (25.4) |  |
| 25–29.9 | 165 (83.3) | 33 (16.7) |  |
| ≥30 | 104 (91.2) | 10 (8.8) |  |
| **Education** |  |  | **<0.001** |
| Less than high school | 7 (87.5) | 1 (12.5) |  |
| High school or equivalent | 26 (78.8) | 7 (21.2) |  |
| College Diploma | 107 (87.7) | 15 (12.3) |  |
| Bachelor’s degree | 194 (74.6) | 66 (25.4) |  |
| Master’s degree or higher | 122 (89.7) | 14 (10.3) |  |
| **Zones** |  |  | **0.003** |
| Central Zone | 40 (95.2) | 2 (4.8) |  |
| Dar es salaam | 180 (74.7) | 61 (25.3) |  |
| Lake Zone | 105 (87.5) | 15 (12.5) |  |
| Northern Zone | 62 (82.7) | 13 (17.3) |  |
| Southern Highlands Zone | 34 (82.9) | 7 (17.1) |  |
| Southern Zone | 30 (90.9) | 3 (9.1) |  |
| Zanzibar | 5 (71.4) | 2 (28.5) |  |
| **Residency** |  |  | **0.004** |
| Rural | 94 (92.2) | 8 (7.8) |  |
| Urban | 362 (79.2) | 95 (20.8) |  |
| **Affiliation** |  |  | 0.431 |
| Government Institution | 260 (84.1) | 49 (15.9) |  |
| Private Institution | 130 (77.8) | 37 (22.2) |  |
| Individual Business | 8 (72.7) | 3 (27.3) |  |
| Unemployed | 40 (78.4) | 11 (21.6) |  |
| Retiree | 4 (80.0) | 1 (20.0) |  |
| Other | 14 (87.5) | 2 (12.5) |  |
| **Workplace** |  |  | 0.119 |
| Community health center | 68 (89.5) | 8 (10.5) |  |
| Dispensary | 25 (80.6) | 6 (19.4) |  |
| District Hospital | 68 (88.3) | 9 (11.7) |  |
| Regional Hospital | 46 (78.0) | 13 (22.0) |  |
| Referral and specialized hospital | 73 (76.0) | 23 (24.0) |  |
| National Hospital | 37 (72.5) | 14 (27.5) |  |
| Other | 99 (83.9) | 19 (16.1) |  |
| Unemployed | 40 (78.4) | 11 (21.6) |  |
| **Chronic diseases** |  |  | 0.784 |
| Yes | 65 (83.3) | 13 (16.7) |  |
| No | 391 (81.3) | 90 (18.7) |  |
| **Insurance** |  |  | 0.345 |
| Yes | 349 (81.7) | 78 (18.3) |  |
| No | 102 (82.2) | 22 (17.8) |  |
| Not sure | 5 (62.5) | 3 (37.5) |  |
| **History of COVID-19 infection** |  |  | 0.423 |
| Yes | 144 (82.3) | 31 (17.7) |  |
| No | 266 (82.4) | 57 (17.6) |  |
| Not sure | 46 (75.4) | 15 (24.6) |  |
| **Role** |  |  | **<0.001** |
| Doctor | 222 (82.8) | 46 (17.2) |  |
| Nurse | 135 (89.4) | 16 (10.6) |  |
| Others | 99 (70.7) | 41 (29.3) |  |

Supplement Table 8. Logistic regression of HCWs' vaccine willingness

|  | Estimate | Std.Error | Z value | P value | OR (95%CI) |
| --- | --- | --- | --- | --- | --- |
| **Role** |  |  |  |  |  |
| Doctor | - | - | - | - | ref |
| Nurse | 0.480 | 0.393 | 1.223 | 0.222 | 1.616 (0.761~3.572) |
| Others | -0.638 | 0.318 | -2.007 | **0.045** | 0.528 (0.282~0.986) |
| **Sex** |  |  |  |  |  |
| Male | - | - | - | - | ref |
| Female | -0.281 | 0.261 | -1.074 | 0.283 | 0.755 (0.452~1.262) |
| **Age** |  |  |  |  |  |
| <30 years | - | - | - | - | ref |
| 30-49 years | 0.360 | 0.310 | 1.162 | 0.245 | 1.433 (0.783~2.644) |
| ≥50 years | 1.565 | 1.284 | 1.218 | 0.223 | 4.781 (0.629~144.528) |
| **BMI** |  |  |  |  |  |
| ≤18.5 | - | - | - | - | ref |
| 18.5–24.9 | -0.759 | 0.681 | -1.114 | 0.265 | 0.468 (0.101~1.576) |
| 25–29.9 | -0.400 | 0.702 | -0.569 | 0.569 | 0.671 (0.14~2.374) |
| ≥30 | 0.383 | 0.754 | 0.508 | 0.612 | 1.466 (0.284~5.927) |
| **Education** |  |  |  |  |  |
| Less than high school | - | - | - | - | ref |
| High school or equivalent | 0.043 | 1.249 | 0.035 | 0.973 | 1.044 (0.046~9.679) |
| College Diploma | 0.024 | 1.184 | 0.020 | 0.984 | 1.024 (0.049~7.857) |
| Bachelor’s degree | -0.528 | 1.180 | -0.447 | 0.655 | 0.590 (0.028~4.501) |
| Master’s degree or higher | 0.322 | 1.235 | 0.261 | 0.794 | 1.38 (0.062~11.994) |
| **Zones** |  |  |  |  |  |
| Central Zone | - | - | - | - | ref |
| Dar es salaam | -1.017 | 0.812 | -1.252 | 0.211 | 0.362 (0.053~1.476) |
| Lake Zone | -0.562 | 0.827 | -0.679 | 0.497 | 0.57 (0.082~2.429) |
| Northern Zone | -0.832 | 0.835 | -0.997 | 0.319 | 0.435 (0.062~1.898) |
| Southern Highlands Zone | -0.727 | 0.892 | -0.815 | 0.415 | 0.483 (0.064~2.447) |
| Southern Zone | -0.781 | 0.995 | -0.785 | 0.432 | 0.458 (0.053~3.206) |
| Zanzibar | -2.032 | 1.230 | -1.652 | 0.099 | 0.131 (0.010~1.567) |
| **Residency** |  |  |  |  |  |
| Rural | - | - | - | - | ref |
| Urban | -0.923 | 0.513 | -1.799 | 0.072 | 0.397 (0.134~1.024) |
| **Affiliation** |  |  |  |  |  |
| Government Institution | - | - | - | - | ref |
| Private Institution | -0.817 | 0.320 | -2.551 | **0.011** | 0.442 (0.234~0.824) |
| Individual Business | -1.086 | 0.806 | -1.348 | 0.178 | 0.337 (0.073~1.868) |
| Unemployed | -0.946 | 0.633 | -1.496 | 0.135 | 0.388 (0.109~1.322) |
| Retiree | -1.674 | 1.518 | -1.103 | 0.270 | 0.188 (0.006~5.419) |
| Other | -0.348 | 0.849 | -0.410 | 0.682 | 0.706 (0.156~5.069) |
| **Workplace** |  |  |  |  |  |
| Community health center | - | - | - | - | ref |
| Dispensary | -1.561 | 0.726 | -2.150 | **0.032** | 0.210 (0.049~0.874) |
| District Hospital | -0.354 | 0.577 | -0.614 | 0.539 | 0.702 (0.220~2.168) |
| Regional Hospital | -0.998 | 0.566 | -1.764 | 0.078 | 0.369 (0.117~1.095) |
| Referral and specialized hospital | -0.983 | 0.519 | -1.893 | 0.058 | 0.374 (0.129~1.001) |
| National Hospital | -1.354 | 0.603 | -2.247 | **0.025** | 0.258 (0.076~0.820) |
| Other | -0.146 | 0.518 | -0.282 | 0.778 | 0.864 (0.300~2.328) |
| **Chronic diseases** |  |  |  |  |  |
| Yes | - | - | - | - | ref |
| No | 0.145 | 0.384 | 0.379 | 0.705 | 1.156 (0.560~2.541) |
| **Insurance** |  |  |  |  |  |
| Yes | - | - | - | - | ref |
| No | 0.548 | 0.328 | 1.673 | 0.094 | 1.731 (0.922~3.349) |
| Not sure | -0.451 | 0.830 | -0.543 | 0.587 | 0.637 (0.128~3.645) |
| **History of COVID-19 infection** |  |  |  |  |  |
| Yes | - | - | - | - | ref |
| No | -0.060 | 0.287 | -0.210 | 0.834 | 0.942 (0.532~1.646) |
| Not sure | -0.400 | 0.410 | -0.976 | 0.329 | 0.67 (0.303~1.520) |

Supplement Table 8. Factors associated with HCWs' vaccine hesitancy

| Variables, n (%) | Hesitancy (n=350) | Without hesitancy (n=209) | P value |
| --- | --- | --- | --- |
| **Sex** |  |  | **0.013** |
| Male | 187 (58.1) | 135 (41.9) |  |
| Female | 163 (68.8) | 74 (31.2) |  |
| **Age** |  |  | **<0.001** |
| <30 years | 174 (71.9) | 68 (28.1) |  |
| 30-49 years | 163 (55.6) | 130 (44.4) |  |
| ≥50 years | 13 (54.2) | 11 (45.8) |  |
| **BMI, Kg/m^2^** |  |  | 0.096 |
| ≤18.5 | 10 (43.5) | 13 (56.5) |  |
| 18.5–24.9 | 151 (67.4) | 73 (32.6) |  |
| 25–29.9 | 120 (60.6) | 78 (39.4) |  |
| ≥30 | 69 (60.2) | 45 (39.8) |  |
| **Education** |  |  | **0.004** |
| Less than high school | 5 (62.5) | 3 (37.5) |  |
| High school or equivalent | 22 (66.7) | 11 (33.3) |  |
| College Diploma | 82 (67.2) | 40 (32.8) |  |
| Bachelor’s degree | 175 (67.3) | 85 (32.7) |  |
| Master’s degree or higher | 66 (48.5) | 70 (51.5) |  |
| **Zones** |  |  | 0.562 |
| Central Zone | 23 (54.8) | 19 (45.2) |  |
| Dar es salaam | 155 (64.3) | 86 (35.7) |  |
| Lake Zone | 70 (58.3) | 50 (41.7) |  |
| Northern Zone | 53 (70.7) | 22 (29.3) |  |
| Southern Highlands Zone | 26 (63.4) | 15 (36.6) |  |
| Southern Zone | 19 (57.6) | 14 (42.4) |  |
| Zanzibar | 4 (57.1) | 3 (42.9) |  |
| **Residency** |  |  | 0.758 |
| Rural | 62(60.7) | 40(39.3) |  |
| Urban | 288 (63.0) | 169 (37.0) |  |
| **Affiliation** |  |  | 0.576 |
| Government Institution | 195 (63.1) | 114 (36.9) |  |
| Private Institution | 109 (65.3) | 58 (34.7) |  |
| Individual Business | 7 (63.6) | 4 (36.4) |  |
| Unemployed | 29 (56.9) | 22 (43.1) |  |
| Retiree | 3 (60.0) | 2 (40.0) |  |
| Other | 7 (43.8) | 9 (56.3) |  |
| **Workplace** |  |  | 0.268 |
| Community health center | 46 (60.5) | 30 (39.5) |  |
| Dispensary | 18 (58.1) | 13 (41.9) |  |
| District Hospital | 53 (68.8) | 24 (31.2) |  |
| Regional Hospital | 40 (67.8) | 19 (32.2) |  |
| Referral and specialized hospital | 68 (70.8) | 28 (29.2) |  |
| National Hospital | 31 (60.8) | 20 (39.2) |  |
| Other | 65 (55.1) | 53 (44.9) |  |
| Unemployed | 29 (56.9) | 22 (43.1) |  |
| **Chronic diseases** |  |  | 0.274 |
| Yes | 44 (55.4) | 34 (43.6) |  |
| No | 306 (63.6) | 175 (36.4) |  |
| **Insurance** |  |  | 0.382 |
| Yes | 264 (61.1) | 163 (38.9) |  |
| No | 79 (63.2) | 45 (36.8) |  |
| Not sure | 7 (87.5) | 1 (12.5) |  |
| **History of COVID-19 infection** |  |  | 0.700 |
| Yes | 107 (61.1) | 68 (38.9) |  |
| No | 202 (62.5) | 121 (37.5) |  |
| Not sure | 41 (67.2) | 20 (32.8) |  |
| **Role** |  |  | 0.096 |
| Doctor | 156 (58.2) | 112 (41.8) |  |
| Nurse | 98 (64.9) | 53 (35.1) |  |
| Others | 96 (68.6) | 44 (31.4) |  |

Supplement Table 9. Logistic regression of HCWs' vaccine hesitancy

|  | Estimate | | Std.Error | Z value | P value | OR (95%CI) |
| --- | --- | --- | --- | --- | --- | --- |
| **Role** |  | |  |  |  |  |
| Doctor | - | | - | - | - | ref |
| Nurse | -0.042 | | 0.285 | -0.146 | 0.884 | 0.959 (0.548~1.681) |
| Others | 0.242 | | 0.270 | 0.898 | 0.369 | 1.274 (0.753~2.171) |
| **Sex** |  | |  |  |  |  |
| Male | - | | - | - | - | ref |
| Female | 0.376 | | 0.209 | 1.801 | 0.072 | 1.457 (0.969~2.199) |
| **Age** |  | |  |  |  |  |
| <30 years | - | | - | - | - | ref |
| 30-49 years | -0.615 | | 0.250 | -2.465 | **0.014** | 0.541 (0.330~0.879) |
| ≥50 years | -0.669 | | 0.533 | -1.254 | 0.210 | 0.512 (0.179~1.477) |
| **BMI** |  | |  |  |  |  |
| ≤18.5 | - | | - | - | - | ref |
| 18.5–24.9 | 1.035 | | 0.473 | 2.185 | **0.029** | 2.814 (1.119~7.29) |
| 25–29.9 | 1.018 | | 0.484 | 2.106 | **0.035** | 2.769 (1.080~7.312) |
| ≥30 | 0.969 | | 0.496 | 1.955 | 0.051 | 2.637 (1.005~7.133) |
| **Education** |  | |  |  |  |  |
| Less than high school | - | | - | - | - | ref |
| High school or equivalent | -0.056 | | 0.876 | -0.064 | 0.949 | 0.945 (0.153~5.183) |
| College Diploma | -0.062 | | 0.810 | -0.076 | 0.939 | 0.940 (0.170~4.500) |
| Bachelor’s degree | -0.145 | | 0.824 | -0.176 | 0.860 | 0.865 (0.152~4.248) |
| Master’s degree or higher | -0.841 | | 0.858 | -0.980 | 0.327 | 0.431 (0.072~2.267) |
| **Zones** |  | |  |  |  |  |
| Central Zone | - | | - | - | - | ref |
| Dar es salaam | 0.297 | | 0.401 | 0.740 | 0.459 | 1.346 (0.610~2.954) |
| Lake Zone | -0.215 | | 0.397 | -0.542 | 0.588 | 0.806 (0.367~1.752) |
| Northern Zone | 0.411 | | 0.437 | 0.939 | 0.348 | 1.508 (0.639~3.567) |
| Southern Highlands Zone | 0.014 | | 0.490 | -0.028 | 0.978 | 0.986 (0.378~2.593) |
| Southern Zone | -0.263 | | 0.505 | -0.519 | 0.604 | 0.769 (0.285~2.082) |
| Zanzibar | 0.043 | | 0.885 | 0.049 | 0.961 | 1.044 (0.184~6.533) |
| **Residency** |  | |  |  |  |  |
| Rural | - | | - | - | - | ref |
| Urban | -0.085 | | 0.291 | -0.290 | 0.772 | 0.919 (0.516~1.622) |
| **Affiliation** |  | |  |  |  |  |
| Government Institution | - | | - | - | - | ref |
| Private Institution | -0.087 | | 0.248 | -0.350 | 0.726 | 0.917 (0.564~1.491) |
| Individual Business | 0.076 | | 0.731 | 0.104 | 0.917 | 1.079 (0.266~4.931) |
| Unemployed | -0.516 | | 0.445 | -1.158 | 0.247 | 0.597 (0.248~1.428) |
| Retiree | 0.782 | | 1.063 | 0.735 | 0.462 | 2.185 (0.279~21.249) |
| Other | -0.776 | | 0.576 | -1.347 | 0.178 | 0.460 (0.144~1.419) |
| **Workplace** |  | |  |  |  |  |
| Community health center | - | | - | - | - | ref |
| Dispensary | -0.232 | | 0.488 | -0.476 | 0.634 | 0.793 (0.304~2.079) |
| District Hospital | 0.541 | | 0.373 | 1.451 | 0.147 | 1.718 (0.829~3.592) |
| Regional Hospital | 0.500 | | 0.405 | 1.235 | 0.217 | 1.649 (0.749~3.683) |
| Referral and specialized hospital | 0.726 | | 0.371 | 1.958 | **0.050** | 2.067 (1.002~4.305) |
| National Hospital | 0.162 | | 0.451 | 0.360 | 0.719 | 1.176 (0.487~2.866) |
| Other | 0.171 | | 0.351 | 0.487 | 0.626 | 1.186 (0.595~2.364) |
| **Chronic diseases** |  | |  |  |  |  |
| Yes | - | | - | - | - | ref |
| No | -0.350 | | 0.280 | -1.250 | 0.211 | 0.704 (0.407~1.224) |
| **Insurance** |  | |  |  |  |  |
| Yes | - | | - | - | - | ref |
| No | <0.001 | | 0.265 | -0.001 | 0.999 | 1.000 (0.596~1.690) |
| Not sure | 0.754 | | 1.137 | 0.663 | 0.507 | 2.125 (0.324~43.038) |
| **History of COVID-19 infection** | |  |  |  |  |  |
| Yes | - | | - | - | - | ref |
| No | -0.066 | | 0.218 | -0.302 | 0.763 | 0.936 (0.609~1.434) |
| Not sure | 0.200 | | 0.340 | 0.589 | 0.556 | 1.222 (0.632~2.409) |

1. [↑](#footnote-ref-1)
